# Gestational Protein Restriction Promotes Osteoporotic Phenotype During Aging

**DOI:** 10.1007/s00223-026-01586-8

**Published:** 2026-07-24

**Authors:** Bruno Calsa, Saul Lindo-Samanamud, Giovani Vinhato Breanza, Renata Marinho Melo, José Antônio Rocha Gontijo, Milton Santamaria-Jr, Patrícia Aline Boer

**Affiliations:** 1 Fetal Programming and Hydroelectrolyte Metabolism Laboratory, Department of Internal Medicine, School of Medical Sciences, Campinas State University (UNICAMP), Campinas, SP Brazil; 2 Brazilian Biosciences National Laboratory (LNBiO), Brazilian Center for Research in Energy and Materials (CNPEM), Campinas, SP Brazil; 3 Departament of Medical Genetics, School of Medical Sciences, Campinas State University (UNICAMP), Campinas, SP Brazil; 4 Department of Social and Paediatric Dentistry, School of Dentistry, Institute of Science and Technology (ICT), São Paulo State University (UNESP), São José dos Campos, São Paulo, Brazil; 5 Department of Dental Materials and Prosthesis, School of Dentistry, Institute of Science and Technology (ICT), São Paulo State University (UNESP), São José dos Campos, São Paulo, Brazil; 6 National Institute of Science and Technology in Developmental Origins of Health and Disease (INCT-DOHaD Brasil), Campinas, São Paulo Brazil

**Keywords:** DOHaD, Bone loss, Immunoporosis, Femur

## Abstract

Osteoporosis results from impaired bone remodeling and deterioration of bone microarchitecture, ultimately increasing fracture risk. Although early-life nutritional stress has been implicated in long-term health outcomes, its impact on skeletal remodeling mechanisms remains unclear. We investigated whether gestational protein restriction induces persistent alterations in the skeletal immune microenvironment that contribute to age-related bone fragility. Pregnant C57BL/6J mice were fed either a normal-protein or low-protein (LP) diet during gestation. Male offspring were evaluated at 12 and 18 months of age. Microcomputed tomography revealed significant reductions in trabecular bone volume fraction, bone mineral density, and trabecular number in 18-month-old LP offspring, along with reduced cortical thickness at both ages. Three-point bending tests demonstrated reduced maximal load in LP-18 M femora, indicating compromised mechanical strength. Ultrastructural analyses revealed increased cortical porosity and disrupted collagen fiber organization in aged LP bone. RNA sequencing identified differentially expressed genes enriched in pathways related to macrophage recruitment, complement signaling, and osteoclast differentiation. RT-qPCR confirmed increased expression of *C5ar1*, *Ccl3*, *Ccr2*, *Lilrb4a*, and *Trem2*. Immunohistochemical analysis demonstrated increased CD68^+^ cells in aged LP animals, indicating expansion of the skeletal myeloid compartment. These findings demonstrate that gestational protein restriction induces long-term reprogramming of the skeletal immune microenvironment, associated with enhanced osteoclast-related signaling and impaired bone mechanical competence during aging.

**Supplementary Information:**

The online version contains supplementary material available at 10.1007/s00223-026-01586-8.

## Introduction

Osteoporosis is a chronic skeletal disorder marked by reduced bone mineral density (BMD), compromised bone microarchitecture, and increased susceptibility to fractures, especially in elderly individuals. The condition represents a global health issue due to its association with age-related fragility fractures, which lead to significant morbidity, mortality, and healthcare cost [1–3]. Although osteoporosis is commonly associated with hormonal decline, sedentary lifestyle, and aging, early-life environmental exposures are increasingly recognized as critical contributors to lifelong skeletal health [4–6].

As the organism aged, multiple processes contribute to the progression of bone fragility. Among them, chronic low-grade inflammation plays a central role by promoting osteoclast (OCs) differentiation and activation, often through TNF and chemokine signaling pathways [7]. Enhanced osteoclastic bone resorption disrupts the balance of bone remodeling, that could accelerate bone loss. Additionally, structural alterations in extracellular matrix, such as collagen fiber disorganization, compromise the mechanical integrity of bone [8]. Aging is associated with reduced collagen-mineral interactions and impaired intra- and extrafibrillar mineralization [9] which diminish matrix ability to dissipate mechanical loads. Consequently, the tissue becomes stiffer but more prone to fracture.

One key concept explaining the long-term impact of early-life conditions on adult health is the Developmental Origins of Health and Disease (DOHaD). Prenatal nutritional status plays a crucial role in programming organ systems, including the skeletal system. Among various models of intrauterine insults, gestational protein restriction (GPR) has been widely used in rodents to investigate how maternal undernutrition influences postnatal outcomes. Beyond craniofacial complex, we have reported that maternal insults affect craniofacial development, particularly in the mandible and maxilla [10–12] and teeth [13, 14]. Mandibles from low-protein offspring exhibit reduced mineralization near the end of gestation, as well as significant alterations in the expression of genes involved in osteoblast differentiation [12]. However, the long-term effects of GPR on skeletal aging and osteoporosis susceptibility remain unexplored.

Despite evidence connecting gestational malnutrition to developmental skeletal alterations, there is a notable gap in understanding how these early-life insults affect bone quality and physiology in later life, particularly in the context of aging. To address this, the present study investigates the long-term consequences of gestational low-protein diet on bone and we hypothesized that gestational protein restriction predisposes offspring to an osteoporosis-like phenotype in aging, driven by inflammatory activation, osteoclast-mediated bone resorption, and matrix disorganization.

## Materials & Methods

### Animal Ethics

All procedures were performed in accordance with the Ethical Principles of Animal Experimentation adopted by the National Council for the Control of Animal Experimentation (CONCEA) and were approved by the Ethics Committee (#5923-1/2021 - UNICAMP) and ARRIVE (Animal Research: Reporting of In vivo experiments) guideline.

### Experimental Model

The C57BL/6J mice were maintained in an environment with a temperature of 23 ± 2 °C, relative humidity of 50 ± 10%, and a light-dark cycle of 12 h. Mating occurred during the dark cycle. Upon confirmation of a vaginal plug, pregnant mice were housed individually and assigned to dietary groups: control group received a normal-protein (NP, *n* = 10) diet containing 17% protein, while the protein-restricted group received a low-protein (LP, *n* = 10) diet containing 6% protein throughout gestation [12, 15, 16].

One day after birth, pups were weighed, and all litters were transitioned to a standard diet (Purina Labina^®^) and water *ad libitum* until weaning. After weaning, the offspring were housed and maintained until the designated euthanasia time points: 12 months of age (12 M), representing middle-aged mice; and 18 months of age (18 M), representing elderly mice. For femur collection, animals were anesthetized using 3% of isoflurane in oxygen and the left femur were cleaned for soft tissue and processed according to assessment requirement.

### Ex Vivo Microtomography Computed

The left femurs were fixed in 4% paraformaldehyde (PFA) during 24 h and stored in ethanol 70%. Prior scanning, the tissue was hydrated in phosphate buffer for 24 h. Samples (*n* = 4/group/age) were imaged at SkyScan 1272 microCT system (Bruker, Kontich, Belgium) in three spatial planes using the following parameters: 70 kVp, 142 µA, 2000 ms exposure time, and a voxel size of 9 microns. Tomographic sections were reconstructed using NRecon software (Bruker). For trabecular bone analysis, 100 consecutive slices were acquired from the distal metaphysis of the femur. The growth plate was identified in the longitudinal orientation, and the region of interest (ROI) was defined immediately distal to its end. Only trabecular bone within this ROI was included, with cortical bone explicitly excluded. For cortical bone parameters, 200 slices were analyzed from the diaphysis. Bone mineral density (BMD) was determined using CTAn software, calibrated with calcium hydroxyapatite phantoms (0.25 and 0.75 g/cm^3^). All parameters were reported according to the guidelines of the American Society for Bone and Mineral Research (ASBMR) for microCT analysis of rodent bone [17].

### Biomechanical Testing

Left femurs (*n* = 4/group/age) were evaluated for mechanical load to failure using three-point bending test on a universal testing machine (EMIC - São José dos Pinhais, PR, Brazil) equipped with a 10 kgf load cell. Each bone was placed horizontally on two support rollers with a span length of 7 mm. A vertical load was applied at the mid-diaphysis on the posterior surface under a constant displacement rate of 1 mm/min until fracture occurred. From the resulting load-displacement curves, the following structural parameters were determined: ultimate force (maximal load, expressed in Newtons, N), defined as the peak force sustained by the femur before failure; structural stiffness (expressed in N/mm), calculated as the slope of the linear elastic region of the curve; and ultimate displacement (expressed in mm), representing the total deformation of the bone up to the fracture point.

### Fracture Assessment

Following mechanical testing, fracture and cortical surfaces were analyzed by scanning electron microscopy (SEM) to assess surface morphology. Energy Dispersive X-Ray Spectroscopy (EDS) analyses were conducted in Map Mode with an accelerating voltage of 10 kV, a field width of 1 mm, using a Secondary Electron Detector (SED). Data acquisition and processing were carried out using the Phenom User Interface (version 2.0.2-rel). Samples were imaged using a Phenom XL G2 microscope (Thermo Fisher Scientific).

### RNA-Seq Bulk

For the RNA-seq bulk experiment, left femur from 18 M animals (*n* = 3/group) was dissected, clean from soft tissue, pulverized in liquid nitrogen, and subjected to RNA extraction using TRIzol reagent. A total of 1 µg of RNA from each sample was used for library preparation with the TruSeq Stranded mRNA Library Prep Kit (Illumina) following manufacture recommendations. Briefly, Poly-A RNA was captured using oligo-dT magnetic beads, then fragmented and converted into first- and second-strand cDNA. Following end repair, A-tailing, and adapter ligation, the libraries were amplified by 15 cycles of PCR. Fragment size distribution was assessed with the Agilent 4200 TapeStation. Sequencing was conducted at the Life Sciences Core Facility (LaCTAD) from State University of Campinas (UNICAMP) on NextSeq 2000 platform, producing an average of 40 million reads per sample.

### Bioinformatics Analysis

Raw reads were trimmed using Trimmomatic v0.39, retaining an average of 85.4% of reads per library for downstream analysis. Clean reads were aligned to *Mus musculus* reference genome (Ensembl release 112) using Bowtie2 v2.4.4, and gene expression levels were quantified using RSEM v1.3.3. Differential expression analysis was performed with edgeR v3.42.4. Genes with FDR < 0.05 (False discovery ratio) were selected for functional enrichment analysis using the clusterProfiler *v4.16.0* R package [18]. GO enrichment was performed for Biological Process (BP) using the enrichGO function, with Benjamini-Hochberg correction and significance thresholds of p-adjusted < 0.05 and q-value < 0.05. The graphs were generate using SRplot.

### Immune Cell Infiltration Analysis

The abundance of 24 immune cell types was estimated using the ImmuCellAI web platform [19]. The analysis was performed using the FPKM dataset. Statistical analyses and data visualization were conducted in R. To compare immune cell abundance between groups, a Student’s t-test was performed. Data are presented as mean ± standard deviation (SD). Heatmaps were generated using the pheatmap package based on Z-score scaled data to visualize relative abundance patterns across samples. Box plots illustrating the distribution of immune scores and specific cell abundances were created using the ggplot2 and ggpubr packages.

### RTqPCR

To validate RNAseq, we performed RTqPCR from selected genes in 12 and 18 M animals (*n* = 3/group/age). Briefly, RNA was extracted using TRIzol reagent and cDNA synthesized using High-Capacity kit (Cat. No. 4368814 - Applied Biosystems). Primers were designed using Primer-BLAST (NCBI) (Table S1). The qPCR cycle was performed using ReadyMix JumpStart™ Taq SYBR^®^ Green (Cat. No. S4438 – Sigma Aldrich) on StepOne Plus system under the following cycle: 95 °C for 2 min, followed by 40 cycles of 95 °C for 15 s and 60–63 °C for 1 min. The gene expression level was calculate using 2^−ΔΔCT^ and data normalization using *Bact* and *Gapdh*.

### Tissue Process for Collagen Quantification

Right femurs (*n* = 4/group/age) were dissected, cleaned of soft tissue, and fixed in PFA 4% at 4 °C for 24 h. After fixation, samples were decalcified in EDTA (627 mM, pH 7.4) for 2–3 weeks with regular solution changes. Following decalcification, bones were cryoprotected in 30% sucrose in PBS at 4 °C for 24 h, embedded in Optimal Cutting Temperature (OCT) compound, frozen in hexane cooled by liquid nitrogen and stored at − 80 °C. Longitudinal cryosections (40 microns) were then obtained and mounted on glass slides.

### Collagen Fibers Assessment

Second Harmonic Generation (SHG) is a label-free optical process, in which two photons combine to produce a new photon with half the wavelength. This phenomenon occurs only in non-centrosymmetric structures and, in biological tissues, selectively highlights organized structures such as type I collagen [20].

SHG and Two-Photon Excited Fluorescence (TPEF) imaging were performed simultaneously in LSM 780-NLO confocal microscope (Carl Zeiss) at the National Institute of Science and Technology on Photonics Applied to Cell Biology (INFABIC). Excitation was provided by Chameleon Discovery NX laser (Coherent) tuned to 940 nm at 100 mW, and focused through 40x/1.3 NA EC Plan-Neofluar oil-immersion objective. Emission signals were separated using filter sets: bandpass (500–550 nm), long-pass (490 nm), and short-pass (485 nm). Image acquisition were acquired with 1024 × 1024 pixels resolution (215 × 215 microns). Collagen fibers quantification was performed using ImageJ software. Briefly, the bone matrix was selected in the 2-PEF channel - periosteum and endosteum was excluded - and the percentage of fibers were quantified.

### Tissue Process for Paraffin Section

Right femurs from 18 M animals (*n* = 5/group) were dissected, cleaned of soft tissue, and fixed in PFA 4% at 4 °C for 24 h. After fixation, samples were decalcified in EDTA (627 mM, pH 7.4) for 2–3 weeks with regular solution changes. Following decalcification, bones were immersed in crescent-ethanol-series and included in Paraplast (Sigma). Serial five-microns sections were performed for hematoxylin and eosin stain, and immunohistochemistry.

### Immunohistochemistry

Sections were deparaffinized and antigen retrieval was conducted in citrate buffer (pH 6.0). The sections were incubated with a blocking solution of 3% bovine serum albumin and incubated overnight at 4 °C with a primary antibody (CD68, 1:200, AbD Serotec – MCA341R). Reactions were developed using DAB (3,3′-diaminobenzidine tetrahydrochloride). After, the slides were dehydrated and mounted with a coverslip. The specimens were photographed in BX51 with DP71 camera (Olympus). Quantification of CD68^+^cells per mm^2^(6 images per animal) were performed in the bone marrow compartment using ImageJ (Multi-point tool). Negative control was showed in Supplementary file.

### Statistical Analysis

Data were assessed for normality using the Shapiro-Wilk test. For normally distributed data, comparisons were made using Student’s t-test, whereas the Mann-Whitney U test was used for non-normal distributions. Statistical analyses were conducted using GraphPad Prism, with significance set at *p* ≤ 0.05. Data are expressed as mean ± standard deviation (SD). The corresponding p-values and sample sizes are indicated in figures.

## Results

### Bone Microarchitecture

**Fig. 1:**
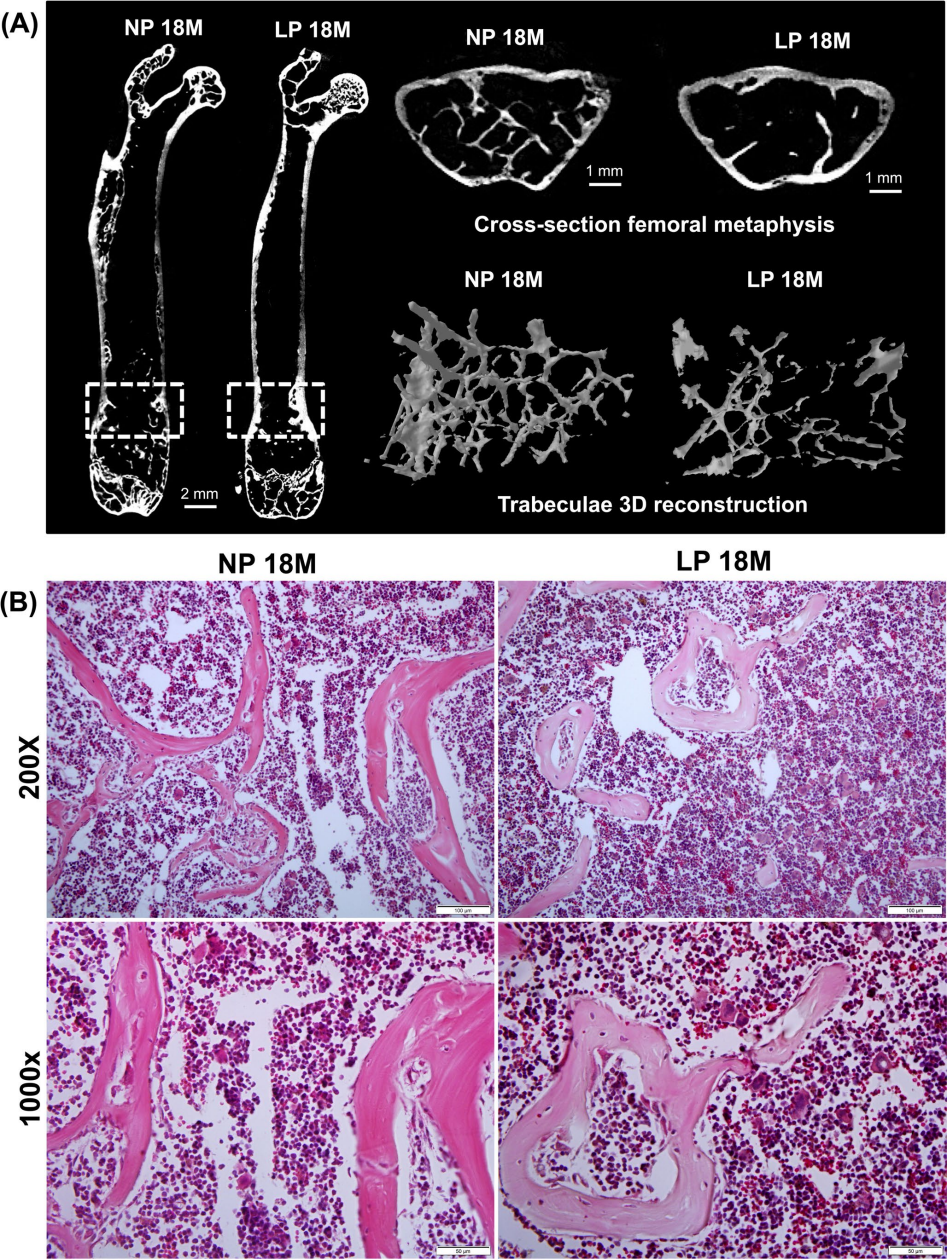
Representative 2D and 3D micro-CT, and hematoxylin and eosin stain of trabecular bone section in distal femur from 18-month-old mice. Gestational protein restriction (LP) results in visible trabecular bone loss compared to controls (NP). Scale bar: 1–2 mm for microCT imagens and 50–100 um for histological images

Representative 2D and 3D reconstructions of micro-CT scans, along with hematoxylin and eosin-stained sections of the distal femur, are shown in Fig. 1. At 18 months of age, offspring exposed to gestational protein restriction (LP) displayed a marked reduction in trabecular bone mass compared to control mice (NP), as evidenced by visible loss of trabecular structures in both micro-CT and histological images. Quantitative analysis of trabecular microarchitecture is summarized in Table 1. At 12 months, LP mice exhibited lower BS/TV, accompanied by increased Tb.Sp, when compared to NP age match-controls. These differences became more pronounced at 18 months, LP mice exhibited lower BV/TV, BS/TV, Tb.Th, Tb.N and BMD, accompanied by increased Tb.Pf and SMI, when compared to NP age match-controls. Cortical bone parameters are presented in Table 2. At 12 months, LP mice exhibited higher cortical surface and lower cortical thickness when compared to NP age match-controls. At 18 months, LP mice exhibited lower cortical thickness and volume when compared to NP age match-controls.

**Table 1:** Microarchitectural parameters of trabecular bone in the distal femur of control (NP) and protein-restricted (LP) mice at 12 and 18 months of age

Parameters	NP	LP	*P* value	
BV/TV (%)	6.25 ± 0.69	9.75 ± 3.80	0.1205	12 months
BS/TV (%)	0.004 ± 0.0002	0.007 ± 0.002	0.0491	
Tb.Th (µm)	61.67 ± 7.56	58.15 ± 2.58	0.4126	
Tb.Sp (µm)	313.0 ± 27.27	252.3 ± 10.4	0.0060	
Tb.N (n/mm)	0.0100 ± 0.0007	0.0100 ± 0.0058	0.0755	
Tb.Pf	0.02574 ± 0.002331	0.02611 ± 0.008524	0.9369	
BMD (mg/cm^− 3^)	13.49 ± 2.947	15.81 ± 4.148	0.3968	
SMI	2.482 ± 0.04653	2.534 ± 0.4800	0.8361	
BV/TV (%)	7.461 ± 1.014	3.755 ± 0.6917	0.0009	18 months
BS/TV (%)	0.005313 ± 0.0007182	0.002433 ± 0.001507	0.0136	
Tb.Th (µm)	61.05 ± 7.752	53.13 ± 2.998	0.0526	
Tb.Sp (µm)	312.2 ± 36.39	409.7 ± 141.1	0.2296	
Tb.N (n/mm)	0.012 ± 0.001	0.005 ± 0.003	0.0083	
Tb.Pf	0.02464 ± 0.002892	0.03846 ± 0.004804	0.0026	
BMD (mg/cm^− 3^)	19.43 ± 1.822	12.56 ± 1.982	0.0022	
SMI	2.463 ± 0.1187	2.869 ± 0.1561	0.0061	

Data are presented as mean ± standard deviation. Parameters include bone volume fraction (BV/TV), bone surface density (BS/TV), trabecular thickness (Tb.Th), trabecular separation (Tb.Sp), trabecular number (Tb.N), trabecular pattern factor (Tb.Pf), bone mineral density (BMD), and structure model index (SMI). Statistical comparisons between NP and LP groups were performed using unpaired t-tests

**Table 2:** Microarchitectural parameters of cortical bone in the medial femur of NP and LP mice at 12 and 18 months of age

Parameters	NP	LP	*P* value	
Cort.V (mm^3^)	2.853 ± 0.14	3.675 ± 0.77	0.0804	12 M
Cort.S (mm^2^)	42.84 ± 5.327	73.05 ± 13.77	0.0093	
Cort.Th (µm)	191.3 ± 2.811	170.1 ± 7.125	0.0014	
Cort.V (mm^3^)	3.867 ± 0.3987	3.032 ± 0.3217	0.0173	18 M
Cort.S (mm^2^)	83.94 ± 24.54	87.83 ± 3.232	0.7637	
Cort.Th (µm)	171.0 ± 5.97	146.3 ± 11.03	0.0076	

Parameters include cortical volume (Cort.V), cortical surface area (Cort.S), and cortical thickness (Cort.Th). Data are shown as mean ± SD, and statistical comparisons were made using unpaired t-tests

### Three-Point Bending Test

**Fig. 2:**
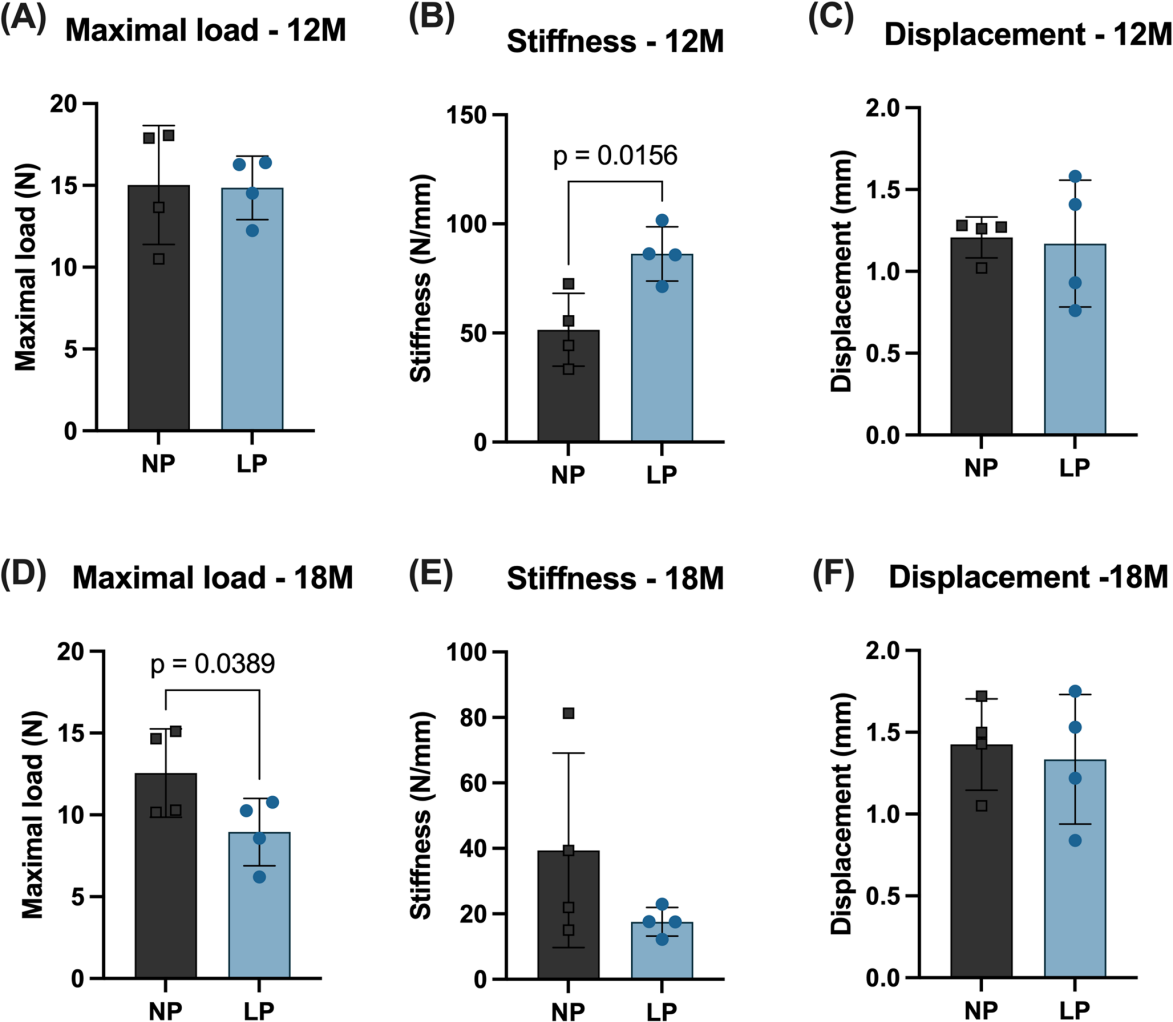
Three-point bending mechanical testing of femurs from NP and LP mice at 12 and 18 months of age. Parameters include stiffness, maximal load, and displacement. Data are presented as mean ± SD. Statistical comparisons were performed using Student’s t-test

To assess the impact of a low-protein diet on bone quality over time, the load to failure, structural stiffness and displacement of femurs from NP and LP mice were evaluated using a three-point bending test at 12 and 18 months of age (Fig. 2). At 12 months, there was no significant difference in the maximal load (Fig. 2A) or displacement (Fig. 2C) between the NP and LP groups. However, femurs from the LP group exhibited significantly higher stiffness compared to those from the NP group (*p* = 0.0156, Fig. 2B). By 18 months, the mechanical integrity of the femurs in the LP group had declined. These mice showed a significantly lower maximal load compared to the age-matched NP group (*p* = 0.0389, Fig. 2D). No significant differences were observed in stiffness (Fig. 2E) or displacement (Fig. 2F) between the two groups at this later time point.

### Fractography and Surface Assessment

**Fig. 3:**
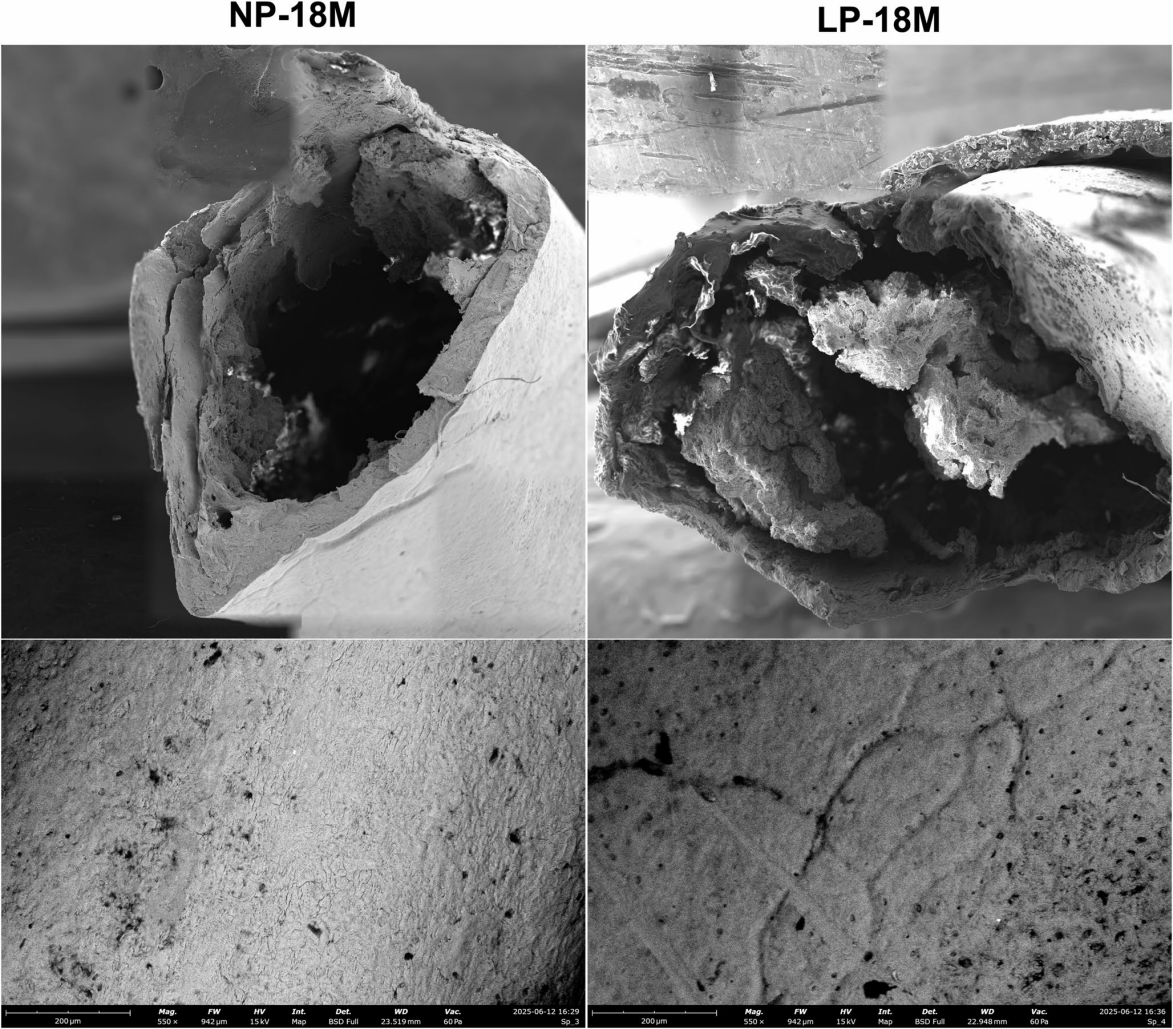
Scanning electron microscopy (SEM) images of the femoral midshaft surface in 18-month-old mice. The LP group exhibits cortical thinning, increased porosity, and surface erosion compared to the NP group. Scale bar: 200 µm; magnification: 550×

To investigate the ultrastructural changes underlying the decline in mechanical strength, the femoral midshafts of 18-month-old mice were examined using scanning electron microscopy (SEM). The results, shown in Fig. 3, reveal clear differences in bone quality between the two groups. Femurs from the NP group displayed a thick cortical shell and a relatively smooth, intact surface, which is characteristic of healthy bone tissue. In contrast, the femurs from the LP group exhibited significant signs of deterioration. The analysis showed notable cortical thinning, increased porosity, and substantial surface erosion with evidence of microcracks. Elemental composition showed no change in fracture surface at 12 and 18-month-old mice, however Ca/S ratio increase in 18 M animals. The results were showed in Supplementary file.

### Collagen Fibers Quantification

**Fig. 4:**
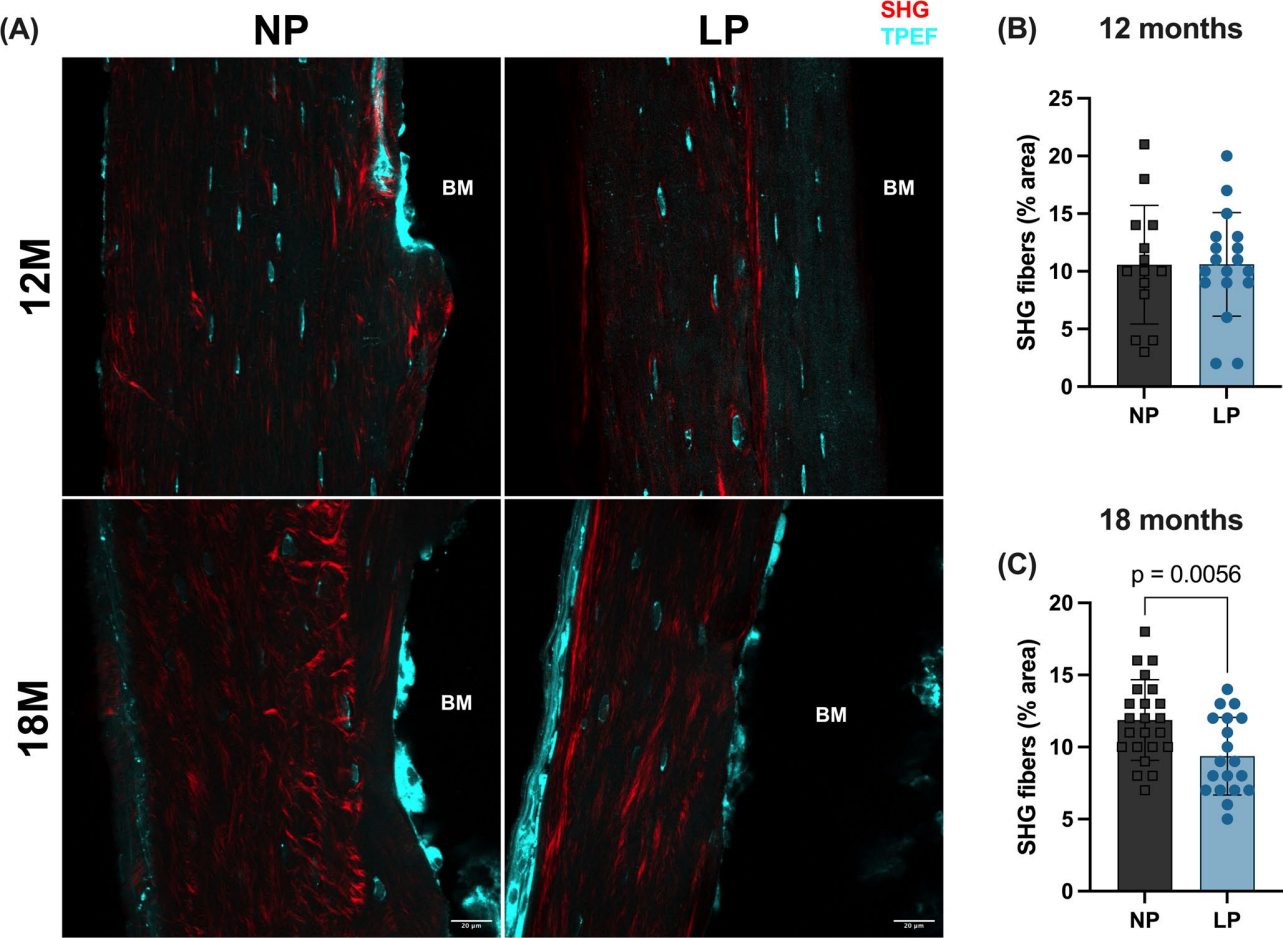
Representative images of collagen fibers (SHG, pseudo-colored red) and autofluorescence signals (TPEF, pseudo-colored cyan) in femoral bone sections from 12M and 18M mice (A). Quantification of collagen fiber percentage in femoral bone at 12 months (B) and 18 months (C). BM: Bone marrow. Scale bar: 20 µm. Data are presented as mean ± SD and analyzed using Student’s t-test.

Collagen fibers were quantified within bone matrix using SHG microscopy and the results showed in Fig. 4. In 12 M animals, no change in SHG fibers were observed between groups (*p* > 0.05). In 18 M animals, SHG fibers reduced in LP group (*p* = 0.0056).

### RNA-Seq

**Fig. 5:**
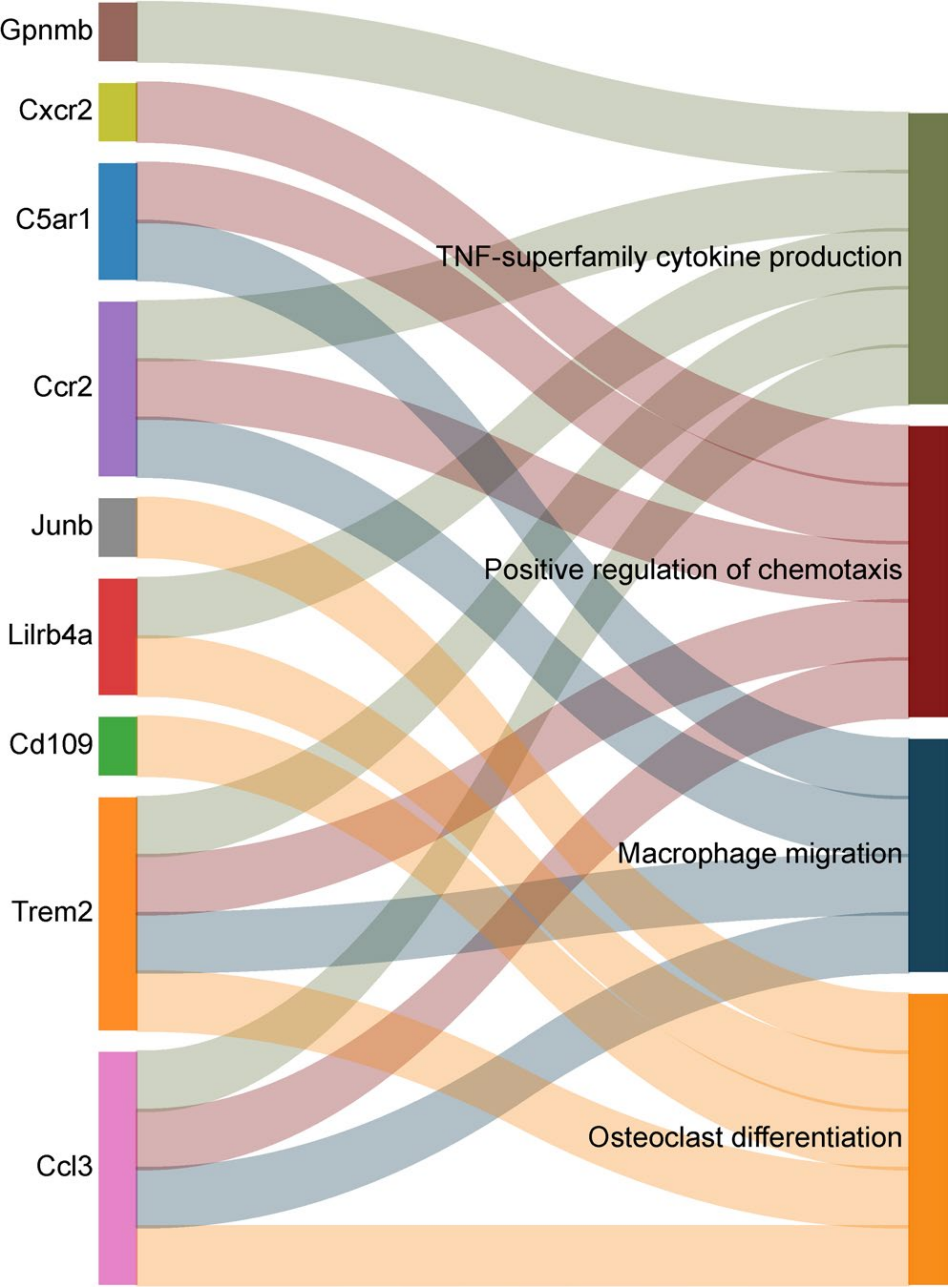
Gene Ontology (GO) enrichment analysis of differentially expressed genes. Biological processes with significant enrichment (FDR < 0.05) are shown. The plot illustrates associations between individual genes (left) and enriched GO terms (right), highlighting pathways such as “osteoclast differentiation”, “TNF superfamily signaling”, and “macrophage migration”. Analysis performed using clusterProfiler and visualized via SRplot

RNA-sequencing analysis revealed distinct transcriptional profiles between the experimental groups. The Principal Component Analysis (PCA) is presented in Supplementary file. Differential expression analysis identified a set of significantly genes, listed in Supplementary Table S2, which served as the basis for subsequent functional enrichment analyses. Functional enrichment analysis (Fig. 5 and Table S3), based on genes with FDR < 0.05, revealed several significantly overrepresented biological processes related to immune and bone remodeling functions: osteoclast differentiation (GO:0030316; q = 0.0101; fold enrichment = 10.22), positive regulation of chemotaxis (GO:0050921; q = 0.0154; fold enrichment = 8.23), tumor necrosis factor superfamily cytokine production (GO:0071706; q = 0.0217; fold enrichment = 6.70), and macrophage migration (GO:1905517; q = 0.0088; fold enrichment = 16.23). Although these pathways were not the most highly represented in terms of gene expression levels, they were prioritized due to their biological relevance and alignment with our central hypothesis involving inflammatory and osteoimmune mechanisms. These enriched terms share key genes such as *Ccl3*, *Trem2*, and *Ccr2*, all of which are known to play pivotal roles in monocyte/macrophage activation and OCs regulation.

#### Immune Cells Estimation

**Fig. 6:**
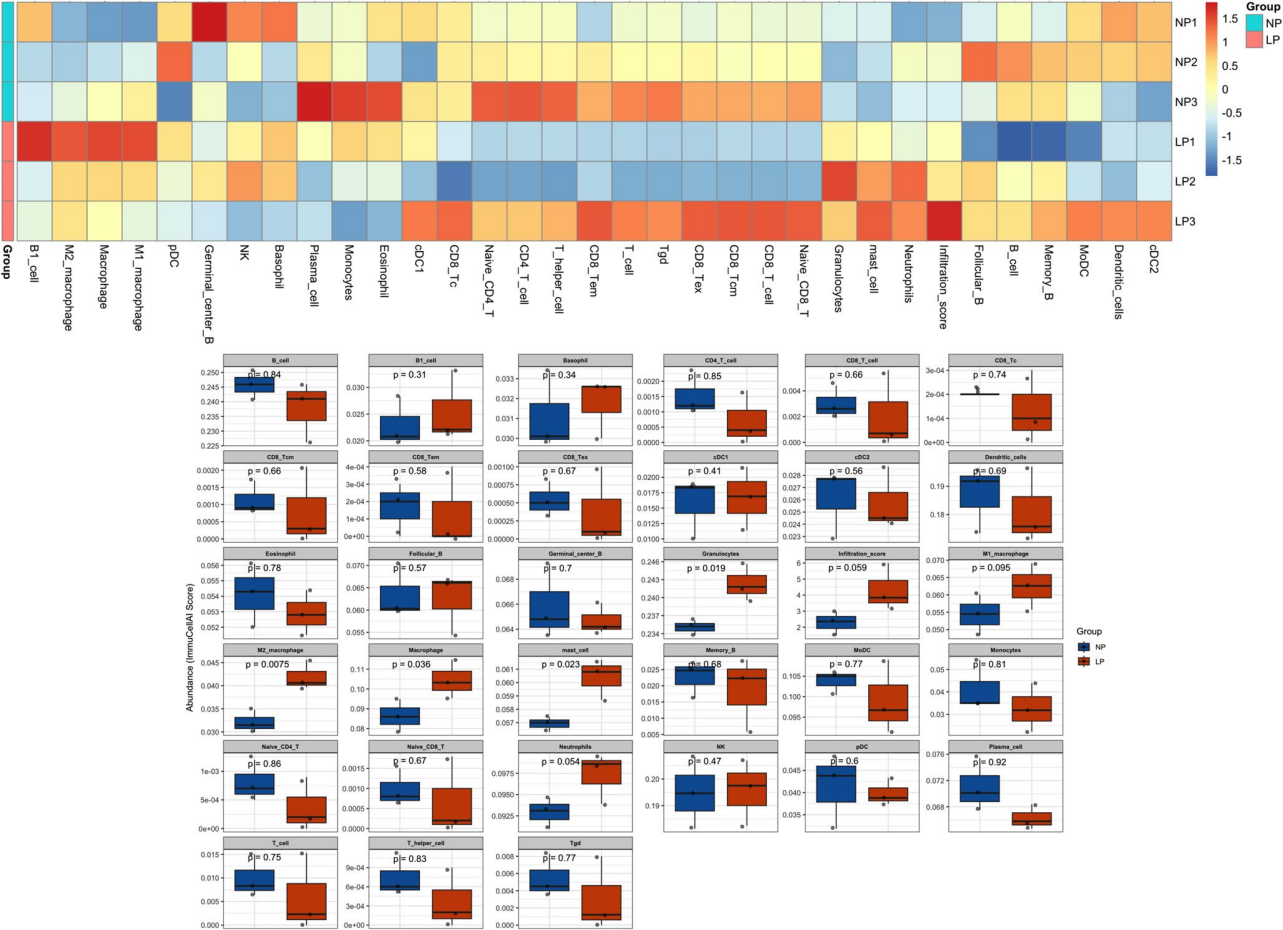
Immune cell infiltration analysis using ImmuCellAI. Data are presented as mean ± SD with individual values. Statistical comparisons were performed using Student’s t-test

Immune cell populations were estimated from the RNA-seq dataset using ImmuCellAI tool. The results were show in Fig. 6. The analysis revealed distinct patterns of immune infiltration between groups, characterized by an increase in macrophages and granulocytes. Further examination of macrophage subsets indicated a significant increase in M2 macrophages in LP animals compared with controls. In granulocytes, were observed an increase in neutrophils and mast cells.

### RTqPCR Validation

**Fig. 7:**
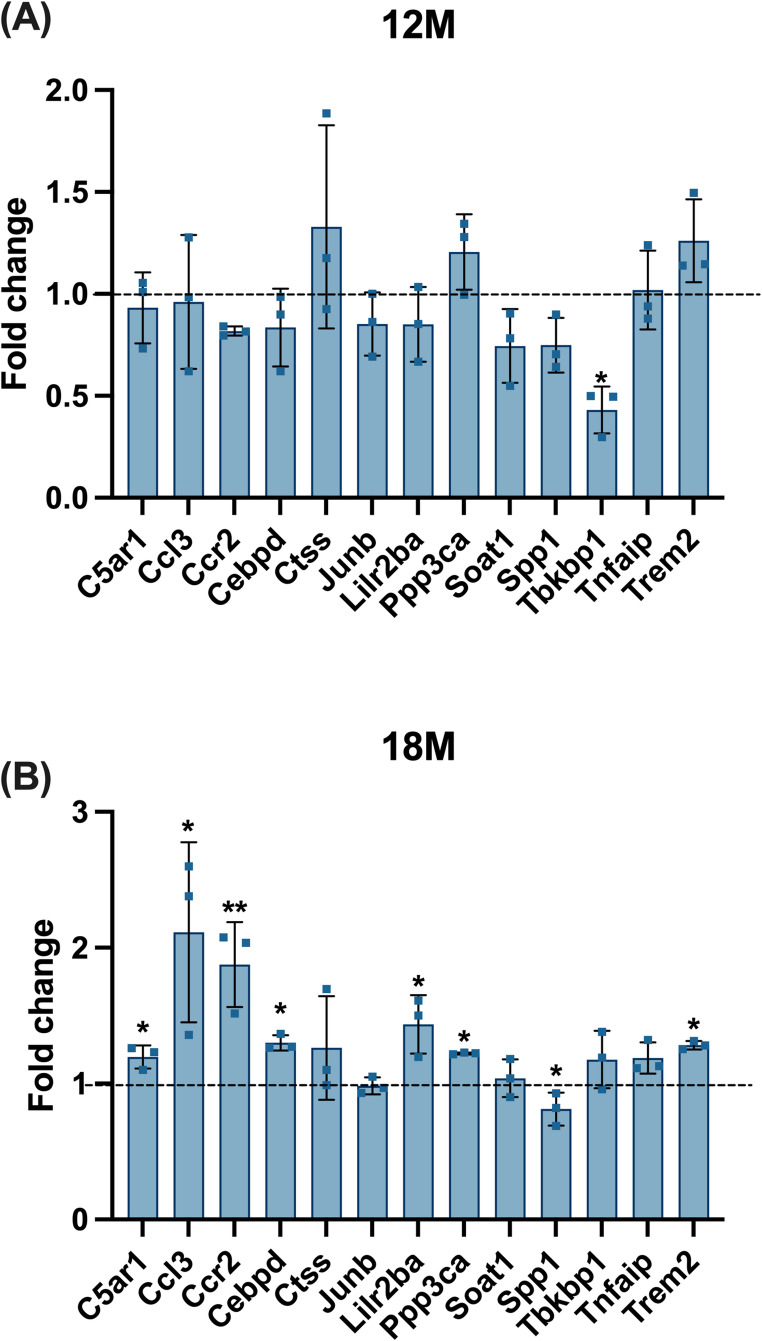
RT-qPCR validation of selected genes involved in bone metabolism in femoral samples from NP and LP mice at 12 and 18 months of age. Relative mRNA expression of C5ar1, Ccl3, Ccr2, Cebpd, Ctss, Junb, Lilrb4a, Ppp3ca, Soat, Spp1, Tbkbp1, Tnfaip, and Trem2 is shown. Expression levels were normalized to Bact and Gapdh. Data are presented as mean ± SD with individual values. Statistical comparisons were performed using Student’s t-test or Mann-Whitney test

RT-qPCR experiments were performed in 12-month-old (12 M) and 18-month-old (18 M) animals to validate selected genes that showed statistical significance (FDR < 0.05) as well as genes with a trend (FDR > 0.05), complementing the enriched biological pathways identified in the RNA-seq analysis. The results were showed in Fig. 7.

### CD68^+^ Cells Quantification

**Fig. 8:**
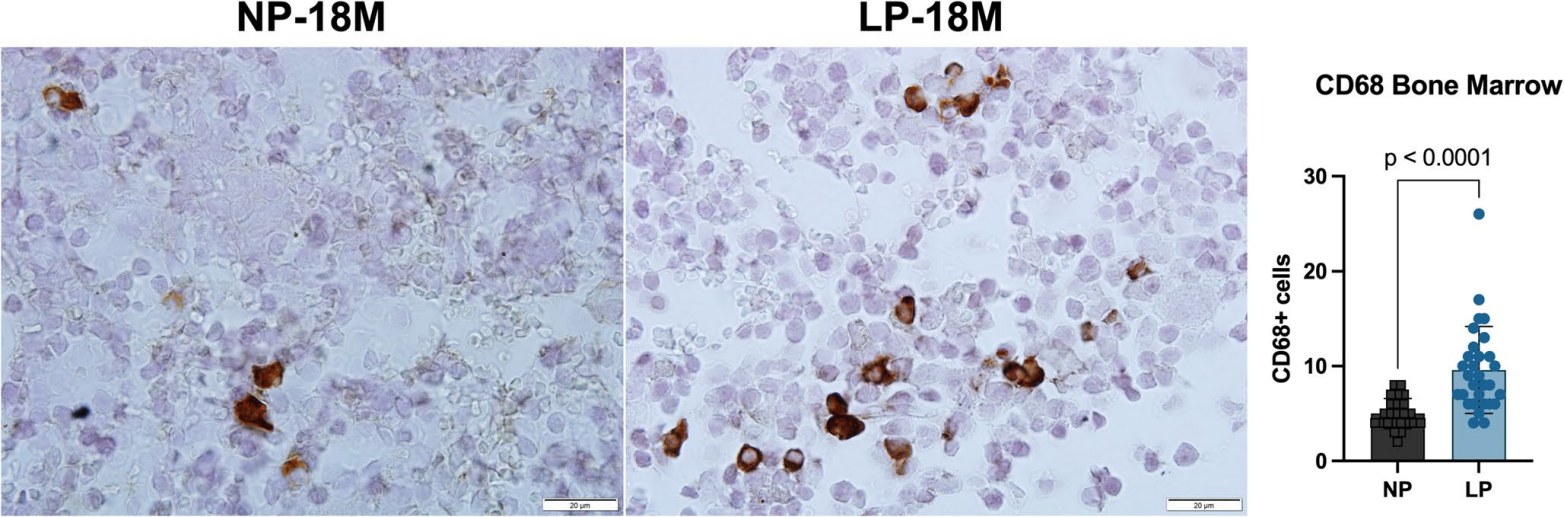
Immunohistochemistry assessment of CD68+ cells within bone-marrow compartment in 18M animals. Data are presented as mean ± SD with individual values. Statistical comparisons were performed using Student’s t-test

To determine if the altered gene expression profile was associated with changes in the bone’s immune cell population, we assessed macrophage presence within the bone marrow. Immunohistochemical staining for the macrophage marker CD68 revealed a notable increase in the number of positive cells in the LP-18 M group compared to the NP-18 M controls. Quantitative analysis confirmed this observation, demonstrating a highly significant increase in the number of CD68^+^ cells in the bone marrow of LP-18 M animals (*p* < 0.0001) (Fig. 8).

## Discussion

This study investigated the long-term effects of GPR on bone physiology in middle-age and aging male offspring. The primary focus was to characterize hallmarks of osteoporosis: bone fragility, reduced mineral density and deterioration of microarchitecture. Our findings demonstrate that intrauterine exposure to a low-protein diet results in both structural and functional impairments in femoral bone, which is evident with advancing age. Furthermore, transcriptomic analysis demonstrates that chronic inflammation as a key mechanism, activating pathways that enhance osteoclast differentiation and promote an osteoporosis-like phenotype.

Our findings demonstrate that animals exposed to GPR exhibit a significant reduction in bone fraction and mineral density at 18 months of age, while no changes are observed at 12 months. This suggests that the effects of GPR on bone health may remain subclinical until later stages of aging. Similar temporal patterns have been reported in intrauterine growth restriction (IUGR) models, leading to compromised trabecular microarchitecture [21]. Consistently, protein restriction during pregnancy and lactation resulted in reduced bone mineral content and density in one-year-old rats [22]. Likewise, intrauterine exposure to a low-protein diet has been shown to cause a 10% reduction in total bone area in adult rats [23].

The absence of bone loss at 12 months reflects the inherent skeletal stability of the C57BL/6 strain, which appears to be resistant to bone loss [24]. In addition, the physiological bone loss in this strain typically initiates only between 16 and 25 months [25–27], the significant BMD reduction observed in 18-month LP animals demonstrates an acceleration of natural senescence. The observed microarchitectural deterioration had direct functional consequences, as bones from 18 M-LP animals presents lower maximum load, suggesting diminished strength. This mechanical failure possibly is attributable to reduction in inorganic and organic content, as evidenced a reduction in SHG-collagen fibers and BMD, respectively.

Clinical studies have established a strong association between low birth weight and an increased risk of osteoporosis later in life [28, 29]. In our experimental model, low birth weight is a consistent and reproducible outcome of GPR [10, 15, 16], reinforcing its validity as a developmental marker of early-life nutritional stress. These findings further support the hypothesis that maternal nutrition plays a critical role in the developmental origins of bone fragility and long-term skeletal health [29]. In this way, the present study provides a stronger data for understanding how an adverse intrauterine environment promotes a higher risk for osteoporotic phenotype.

At the molecular level, RNA sequencing analysis identified 125 differentially expressed genes in the femurs of 18-month-old LP animals. Gene Ontology enrichment revealed a cluster of genes involved in inflammatory processes. Among the enriched biological processes, we highlight OCs differentiation and regulation of macrophage migration. These results suggest the presence of a chronically activated immune microenvironment in the bone tissue of LP animals, which may contribute to increased OCs activity and impaired bone remodeling. These transcriptomic changes suggest that GPR induces pro-inflammatory bone environment during aging, promoting activation of immune cells, particularly macrophages and osteoclasts, which may disrupt bone remodeling homeostasis. The enrichment of pathways related to OCs differentiation further supports the notion that increased bone resorption is a key mechanism driving the osteoporotic phenotype observed in LP animals.

In order to describe cellular mechanisms that promotes the onset of osteoporosis, accumulate evidences coined the term “immunoporosis”, that describe the role of immune cells during osteoporosis [30, 31]. ImmuCellAI analysis revealed an influx of myeloid cells into the bone. For instance, the increased recruitment of myeloid cells, specifically macrophages, granulocytes and mast cells, is likely driven by a distinct chemokine signature, evidenced by the upregulation of the potent monocyte chemoattractant *Ccl3* [32, 33], and the key chemokine receptor *Ccr2* [34]. This influx occurs into a microenvironment primed for inflammation, a state observed by increased expression of the complement receptor *C5ar1*, a known mediator of bone loss [35, 36]. Crucially, once resident in the bone, these infiltrated cells are directed towards a pro-resorptive fate. The significant upregulation of *Trem2*, a pivotal receptor essential for the differentiation, fusion, and survival of osteoclasts, provides a direct link between the immune infiltration and bone degradation [37]. Therefore, we propose that gestational protein restriction programs a lasting, pro-inflammatory state where a specific network of upregulated genes facilitates the recruitment of myeloid precursors and drives their differentiation into bone-resorbing osteoclasts, ultimately leading to the osteoporotic phenotype.

Taken together, our findings demonstrate that gestational protein restriction exerts lasting deleterious effects on femur, with exacerbated consequences in elderly animals. The integrative approach of this study allowed us to characterize this model as an osteoporosis-like phenotype driven by developmental programming.

### Limitations and Future Directions

Despite providing novel results into how GPR programs the femur aging, some limitations of this study should be considered. First, all experiments were performed exclusively in male offspring. We choose to minimize the influence of hormonal fluctuations associated with the female estrous cycle and thereby isolate the long-term effects of gestational protein restriction. However, this approach limits the extrapolation of our findings to females. Future studies including female mice, particularly during estropause.

Second, maintaining animal cohorts until advanced ages (12 and 18 months) presents considerable biological, logistical, and financial challenges. Consequently, the number of animals available for microCT, biomechanical, and transcriptomic analyses was relatively limited (*n* = 3–4 per group). Nevertheless, the sample size was sufficient to detect statistically significant differences between experimental groups across multiple independent endpoints. Moreover, the observed phenotype was consistent among the different analytical approaches.

Third, skeletal characterization was restricted to femur. Although the femur is a widely accepted site for evaluating age-related bone alterations, future studies should investigate additional skeletal regions, particularly trabecular-rich and clinically relevant sites such as the lumbar vertebrae. Such analyses will help determine whether the effects of developmental programming are generalized throughout the skeleton or preferentially affect specific anatomical locations.

In addition, biomechanical assessment was limited to whole-bone structural properties obtained from load–displacement curves, including maximal load, structural stiffness, and displacement. Intrinsic material properties, such as Young’s modulus and tissue-level strength, were not calculated because geometric measurements from the exact fracture site were not integrated with the mechanical testing dataset. Even so, structural biomechanical parameters remain well-established and highly informative indicators of overall bone strength and fracture resistance in murine models [38, 39].

Finally, although bulk RNA sequencing and immunohistochemical analyses identified important inflammatory and osteoclastogenic pathways associated with the observed phenotype, the present study does not establish direct causal relationships between these molecular alterations and skeletal deterioration. Future investigations employing approaches such as single-cell RNA sequencing, pharmacological inhibition of key signaling pathways (e.g., CCR2 or C5aR1), and targeted depletion of myeloid populations will be essential to define the specific cellular mechanisms involved.

## Supplementary Information

Below is the link to the electronic supplementary material.

Supplementary Material 1
