# Supplementary material for: Gestational Protein Restriction Promotes Osteoporotic Phenotype During Aging
Source: Calcif Tissue Int. 2026 Jul 24;117(1):119. doi: 10.1007/s00223-026-01586-8 (PMC13400681; doi:10.1007/s00223-026-01586-8)
Supplement: Supplementary file 1 — Supplementary Material 1 [file 223_2026_1586_MOESM1_ESM.pdf]

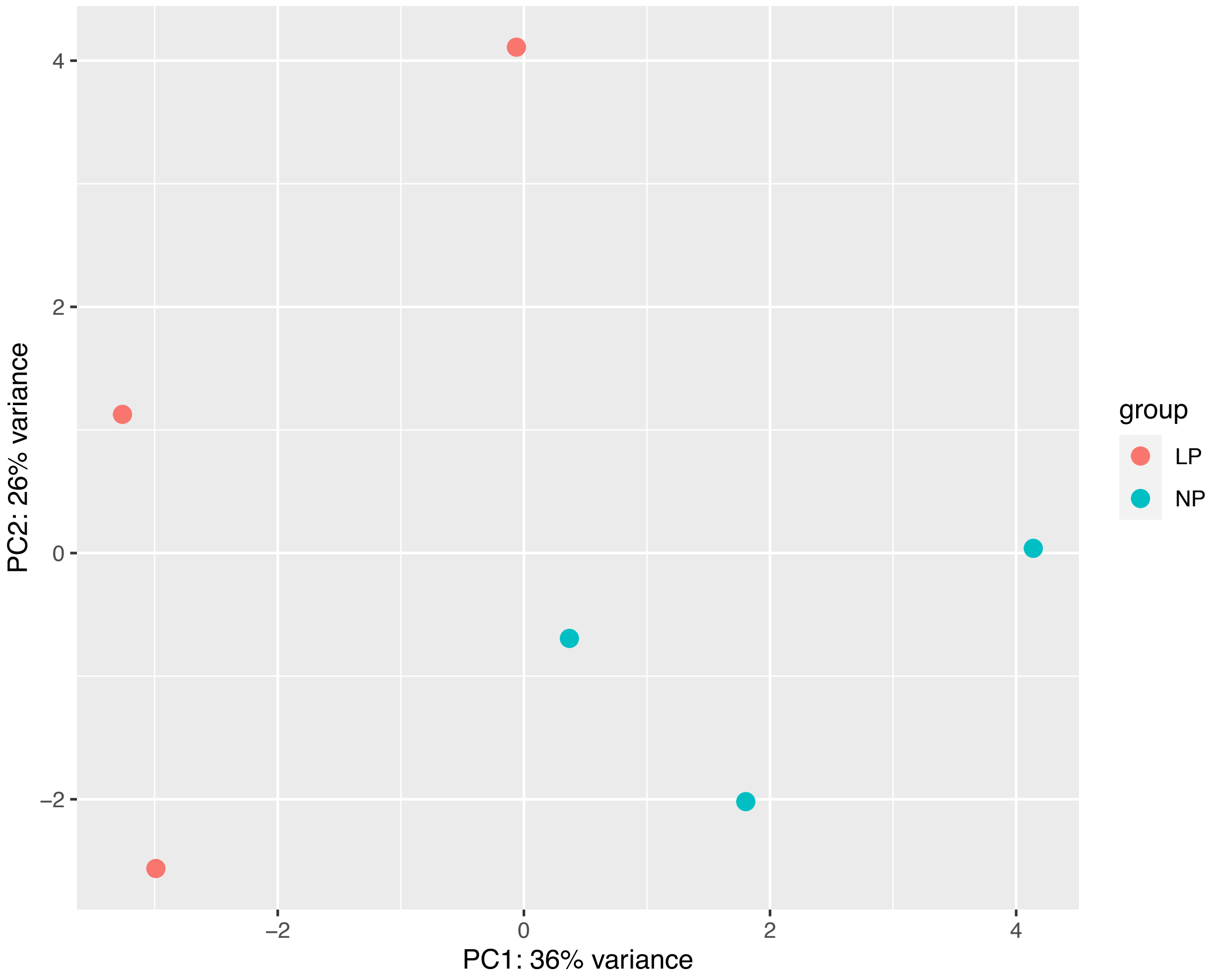

Figure S1. Principal Component Analysis (PCA) of global gene expression. PCA plot displaying the distribution of samples from control (NP) and protein-restricted (LP) groups based on transcriptomic profiles. PC1 explains 36% of the variance and PC2 explains 26%, indicating a clear separation between the two experimental groups.

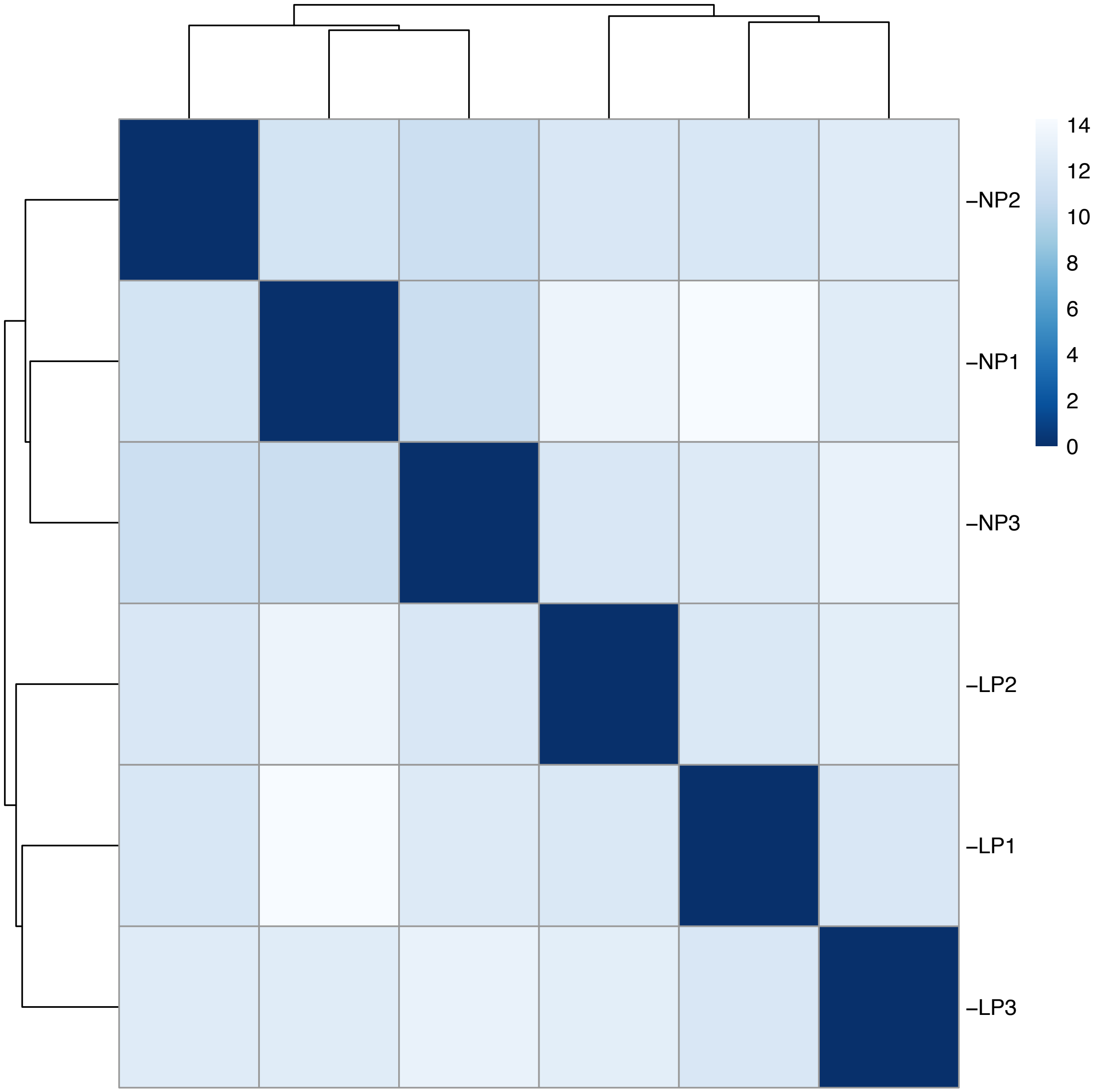

Figure S2. Heatmap of differentially expressed genes. Heatmap showing hierarchical clustering of samples. The analysis reveals clear grouping among biological replicates from the NP (control) and LP (protein-restricted) groups, indicating consistent transcriptomic profiles within each condition.

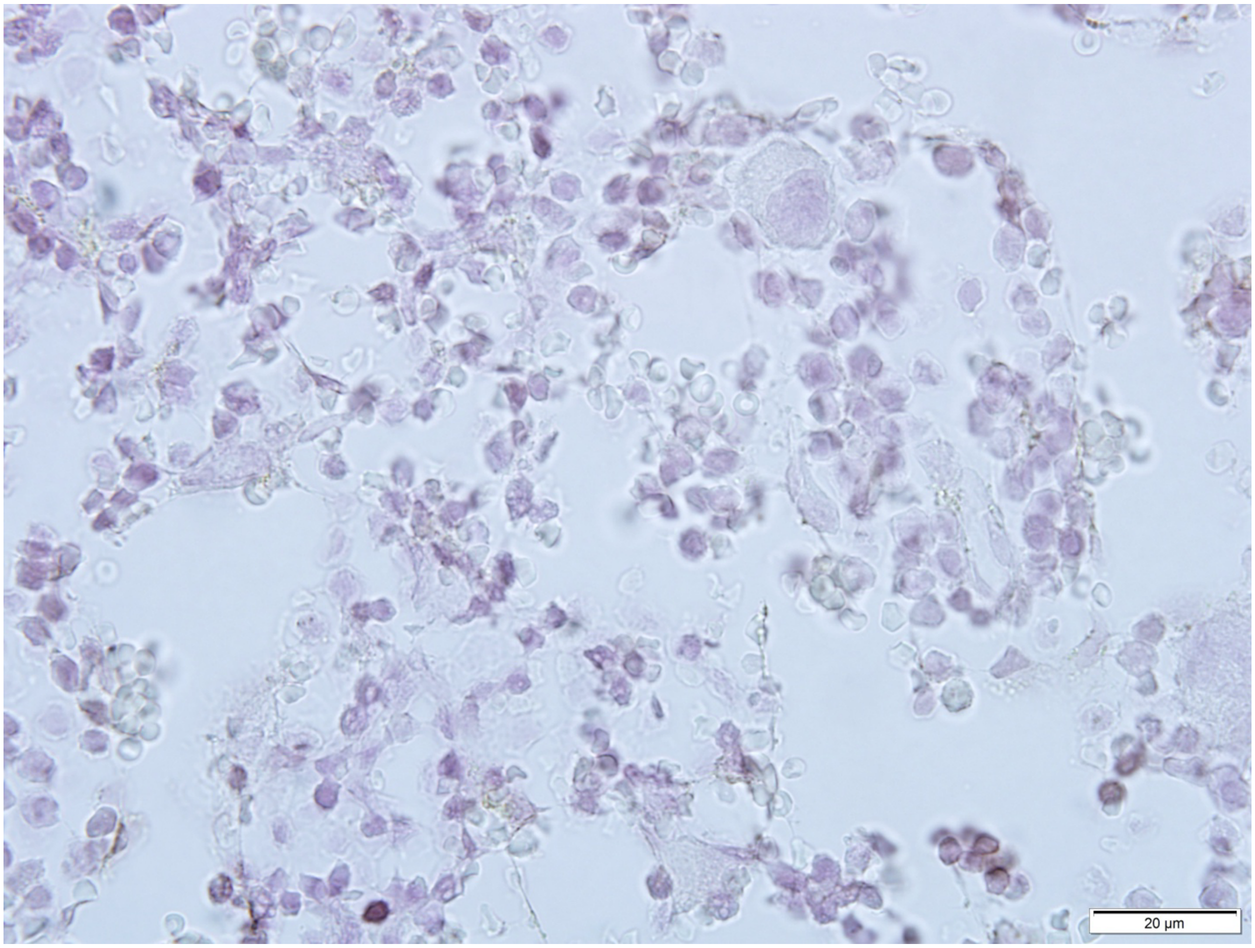

Figure S3: Representative images of negative control reaction. The primary antibody was omitted in sections and counterstained with Haematoxylin. Scale bar: 20 microns.

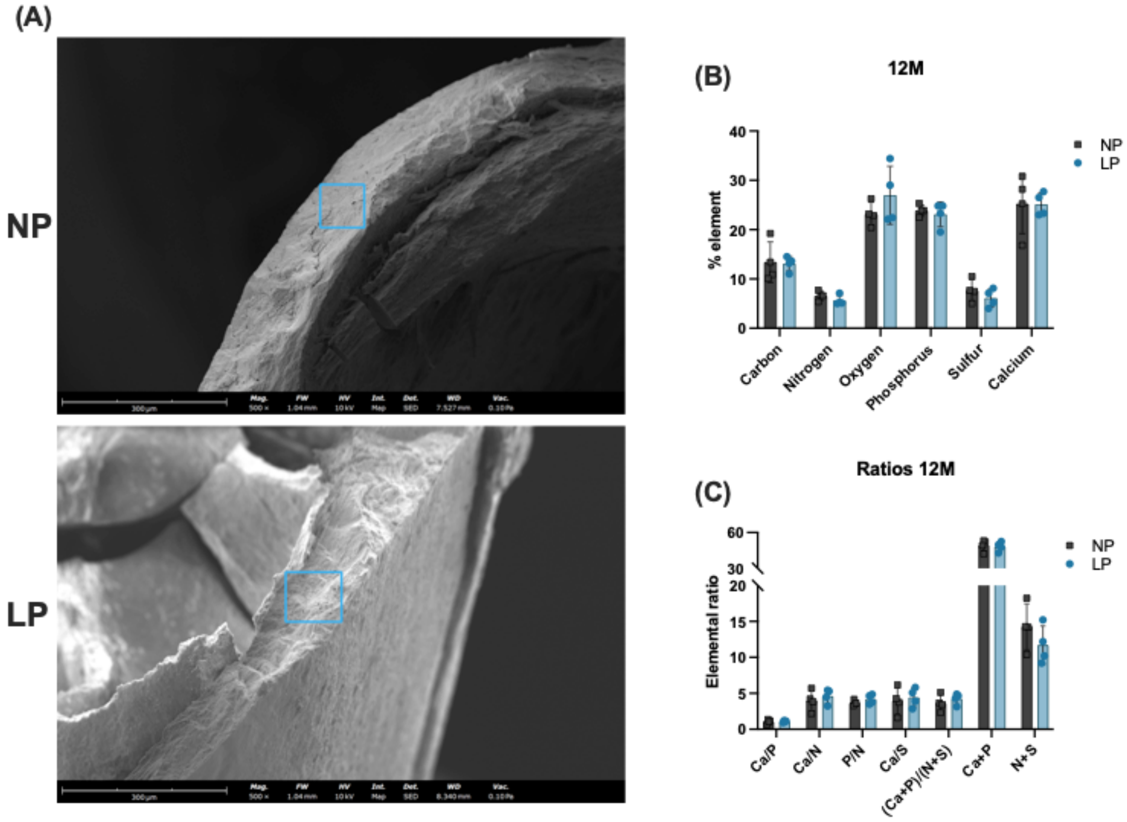

Figure S4: Representative SEM micrographs (500×) and elemental composition of NP and LP groups at 12 months. (A) SEM images showing fracture topography; blue squares indicate the regions of interest (ROI) for EDS analysis. Scale bar = 300 microns. (B) Relative elemental percentages. (C) Calculated elemental ratios. Data are expressed as mean ± SD and compared using Student T test.

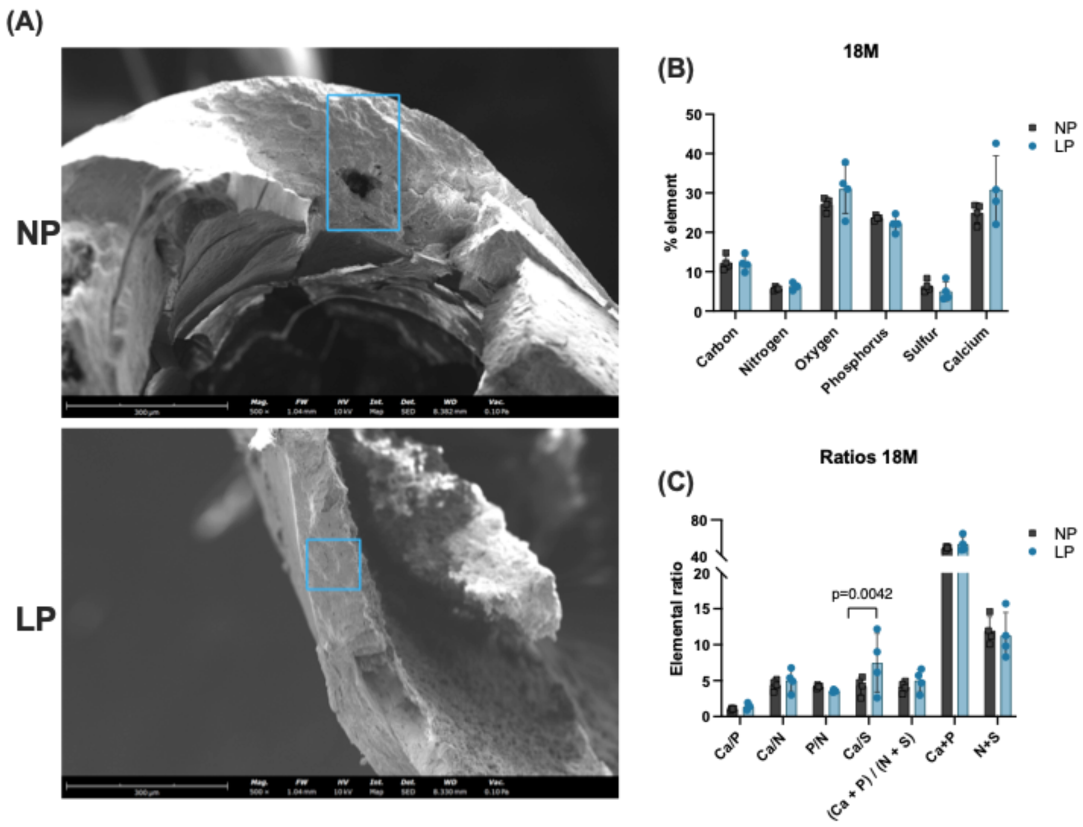

Figure S5: Representative SEM micrographs (500×) and elemental composition of NP and LP groups at 18 months. (A) SEM images showing fracture topography; blue squares indicate the regions of interest (ROI) for EDS analysis. Scale bar = 300 microns. (B) Relative elemental percentages. (C) Calculated elemental ratios; note the significant increase in the Calcium-to-Sulfur (Ca/S) ratio in the LP group ($p=0.0042$), while the Ca/P ratio remains stable. Data are expressed as mean ± SD and compared using Student T test.

Table S1: Primers sequence using for RTqPCR experiments

Gene simbol (NCBI number)	Forward sequence	Reverse sequence
Actb (NM_007393.5)	CACTGTCGAGTCGCGTCC	TCATCCATGGCGAACTGGTG
C5ar1 (NM_007577.4)	GGTTTAAAAGGCACAGCCTGG	TGTTATCTATGGGGTCCATGTCC
Ccl3 (NM_011337.2)	CAGCCAGGTGTCATTTTCCTGA	CCAGGTCTCTTTGGAGTCAGC
Ccr2 (NM_009915.2)	GGAGCCATACCTGTAAATGCC	TGTGGTGAATCCAATGCCCT
Cebpd (NM_007679.4)	ACGACTCCTGCCATGTACGA	TTGAAGAGGTCGGCGAAGAG
Ctss (NM_001267695.2)	CCCTACAAAGCCACGGATGA	ACCAAACGGGAGCTGAATGT
Gapdh (NM_001289726.2)	ACCCTTAAGAGGGATGCTGC	CCCAATACGGCCAAATCCGT
Junb (NM_008416.3)	AGGCAGCTACTTTTCGGGTC	TTGCTGTTGGGGACGATCAA
Lilrb4a/b (NM_001291894.2)	CTGCTATACCTTGGTGAGGTCT	ATGATGGGCTTTGGGAGGTG
Ppp3ca (NM_001428644.1)	GACGCACCAGTCACAGTTTG	AAGAGGTAGCGAGTGTTGGC
Soat1 (NM_009230.3)	TCGCAGGAAGCTTGATTGAT	TTCATGGTAATGTGACCATTTCTG
Spp1 (NM_001204233.1)	ACGACCATGAGATTGGCAGT	CAGTCACTTTCACCGGGAGG
Tbkbp1 (NM_198100.3)	AATCCACCCCTCGCTTTGAG	ATTCGATGGACAGTGGCGTT
Tnfaip8 (NM_001360935.1)	ATGACCGTGTCTGTCTTCGG	GCCAGTGGTTGGGTCTGTTA
Trem2 (NM_001272078.2)	GGAGTCATCGAGTTTCGAGGG	ACAGGATGAAACCTGCCTGGA

gene_id	logFC	logCPM	PValue	FDR	genename
ENSMUSG00000096594	-2,789370288	4,368846624	1,00E-22	1,55E-18	Igkv8-19
ENSMUSG00000094322	-3,604176809	3,154738765	3,88E-18	3,00E-14	Ighv9-4
ENSMUSG00000096078	2,747193776	3,63461427	7,76E-18	3,79E-14	Ighv1-62-2
ENSMUSG00000076596	-1,434677807	2,972047573	9,78E-18	3,79E-14	Igkv3-10
ENSMUSG00000019577	-1,193851245	5,909571011	2,08E-15	6,45E-12	Pdk4
ENSMUSG00000094094	3,423915266	1,936057887	3,29E-13	8,50E-10	Igkv5-45
ENSMUSG00000095335	-1,342718599	3,239043073	3,51E-12	7,76E-09	Igkv3-5
ENSMUSG00000064356	0,685823493	9,135068535	6,01E-11	1,16E-07	mt-Atp8
ENSMUSG00000076655	-1,761300936	2,937764907	8,37E-11	1,44E-07	Ighv4-1
ENSMUSG00000096632	2,151936432	3,616005292	1,07E-10	1,66E-07	Igkv9-124
ENSMUSG00000076552	-1,076594069	3,405416209	1,89E-10	2,67E-07	Igkv4-61
ENSMUSG00000076549	2,546423538	5,853716384	7,24E-10	8,73E-07	Igkv4-68
ENSMUSG00000076594	-1,38063759	2,611452431	7,33E-10	8,73E-07	Igkv6-13
ENSMUSG00000069662	0,418771012	6,278131392	2,92E-09	3,24E-06	Marcks
ENSMUSG00000063415	-1,433241138	2,055514373	7,50E-09	7,74E-06	Cyp26b1
ENSMUSG00000074506	-2,026664332	1,470283477	2,17E-08	2,10E-05	Gm10705
ENSMUSG00000000982	1,727132708	1,150591798	2,46E-08	2,24E-05	Ccl3
ENSMUSG00000096452	1,968544016	2,897616552	2,87E-08	2,47E-05	Ighv1-77
ENSMUSG00000110386	0,457476784	6,402912293	3,87E-08	3,15E-05	Gm42031
ENSMUSG00000101878	-0,469651422	5,290740088	6,79E-08	5,26E-05	Gm8203
ENSMUSG00000021614	1,233009516	3,728188774	1,66E-07	0,000122371	Vcan
ENSMUSG00000096459	-0,726475873	4,546375946	4,27E-07	0,000296707	Ighv9-3
ENSMUSG00000032942	-0,954070949	3,251983751	4,41E-07	0,000296707	Ucp3
ENSMUSG00000023992	0,464466915	4,576090151	5,71E-07	0,000368245	Trem2
ENSMUSG00000000031	-0,739861182	5,571097185	1,21E-06	0,000747769	H19
ENSMUSG00000064358	1,271099052	7,959121467	1,26E-06	0,000753439	mt-Co3
ENSMUSG00000076550	-1,628631565	1,279594248	1,32E-06	0,000758762	Igkv4-63
ENSMUSG00000039062	0,479226243	4,41663946	1,45E-06	0,000782136	Anpep
ENSMUSG00000095571	1,14684273	3,280386285	1,46E-06	0,000782136	Ighv5-17
ENSMUSG00000026832	0,364449215	5,999406141	1,98E-06	0,001022453	Cytip
ENSMUSG00000022157	0,684681312	5,826808573	2,21E-06	0,00110491	Mcpt8
ENSMUSG00000094930	-0,901339454	3,479288427	2,30E-06	0,001114305	Igkv6-25
ENSMUSG00000027533	0,397087164	5,714754066	2,90E-06	0,001359435	Fabp5

ENSMUSG00000095429	-1,854462833	0,970944031	3,42E-06	0,001556284	Ighv5-12
ENSMUSG00000056399	0,381233785	6,707906085	3,94E-06	0,001744722	Prss34
ENSMUSG00000049103	0,338892031	7,083854258	4,25E-06	0,001828589	Ccr2
ENSMUSG00000095127	0,642108828	3,733645678	6,03E-06	0,002523433	Ighv1-82
ENSMUSG00000049562	0,360874452	4,859065917	6,23E-06	0,002539636	Ap5b1
ENSMUSG00000094335	0,640026084	5,086064687	7,13E-06	0,002817541	Igkv1-117
ENSMUSG00000096672	-2,182432739	0,686701699	7,28E-06	0,002817541	Ighv1-63
ENSMUSG00000024480	0,36587499	6,070129711	8,22E-06	0,003041449	Ap3s1
ENSMUSG00000054545	1,121733911	1,601565579	8,26E-06	0,003041449	Ugt1a6a
ENSMUSG00000094088	-0,731488211	3,822055517	8,44E-06	0,003041449	Ighv1-64
ENSMUSG00000076571	2,764255564	0,232087693	8,88E-06	0,003087031	Igkv5-37
ENSMUSG00000112844	2,326529589	-0,456338105	8,97E-06	0,003087031	Gm47036
ENSMUSG00000095416	-1,267088657	2,766189166	9,57E-06	0,003171394	Ighv1-12
ENSMUSG00000057729	0,323076157	8,253246594	9,62E-06	0,003171394	Prtn3
ENSMUSG00000053830	-0,873543825	2,116914953	1,04E-05	0,003370707	Gm9923
ENSMUSG00000076514	1,247483574	2,737059742	1,17E-05	0,00370834	Igkv17-121
ENSMUSG00000047632	-1,605997561	0,270890521	1,66E-05	0,005148799	Fgfbp3
ENSMUSG00000040907	0,314063043	6,714113855	1,94E-05	0,00577748	Atp1a3
ENSMUSG00000001865	0,556843783	4,033230634	1,94E-05	0,00577748	Cpa3
ENSMUSG00000021831	0,270820324	6,281463473	1,98E-05	0,005794721	Ero1a
ENSMUSG00000095351	-1,412124734	4,660561768	2,29E-05	0,006526996	Igkv3-2
ENSMUSG00000030218	0,411261994	5,832624517	2,35E-05	0,006526996	Mgp
ENSMUSG00000055148	0,336553847	5,217233891	2,36E-05	0,006526996	Klf2
ENSMUSG00000043881	0,340146109	5,030764846	2,51E-05	0,006813832	Kbtbd7
ENSMUSG00000037095	0,33967936	6,366696433	2,55E-05	0,006820335	Lrg1
ENSMUSG00000029265	0,289681726	5,655466126	2,78E-05	0,007295334	Dr1
ENSMUSG00000104769	-2,465707224	-0,332412417	3,02E-05	0,007793844	Igkv8-34
ENSMUSG00000110631	0,497548183	7,886428185	3,22E-05	0,007953036	Gm42047
ENSMUSG00000094194	0,929808745	2,676110633	3,23E-05	0,007953036	Ighv5-16
ENSMUSG00000064325	1,670071732	1,813311205	3,23E-05	0,007953036	Hhip
ENSMUSG00000096490	0,9439884	2,923160772	3,40E-05	0,008228453	Igkv10-94
ENSMUSG00000029816	0,471108016	6,228606785	3,58E-05	0,00854347	Gpnmb
ENSMUSG00000039899	0,346332914	5,232752643	3,87E-05	0,009088983	Fgl2
ENSMUSG00000019966	0,352942269	5,276857788	4,27E-05	0,009870946	Kitl

ENSMUSG00000030142	0,367717415	4,491606713	4,54E-05	0,010343134	Clec4e
ENSMUSG00000039270	0,241973993	6,51044697	5,00E-05	0,011231951	Megf9
ENSMUSG00000026600	0,313568764	5,772755408	5,16E-05	0,011421912	Soat1
ENSMUSG00000094102	-1,605838726	1,619678236	5,90E-05	0,012877017	Ighv9-2
ENSMUSG00000028064	0,254304	6,769191874	6,39E-05	0,013745676	Sema4a
ENSMUSG00000074513	0,390015706	4,504963269	7,08E-05	0,015023823	Arfip1
ENSMUSG00000104452	0,868769472	3,185129125	7,38E-05	0,015443829	Ighv8-8
ENSMUSG00000098557	0,267743231	7,533258247	8,28E-05	0,017103261	Kctd12
ENSMUSG00000078122	0,580750424	6,972557183	8,70E-05	0,017724857	F630028O10Rik
ENSMUSG00000039782	0,430394462	4,170328097	9,38E-05	0,018878009	Cpeb2
ENSMUSG00000071637	0,470187596	4,557682505	9,74E-05	0,019352129	Cebpd
ENSMUSG00000030329	1,366333633	0,781463887	0,000104214	0,020435106	Pianp
ENSMUSG00000089901	-0,341130292	5,456084821	0,000108368	0,020984197	Gm8113
ENSMUSG00000000078	0,261433927	6,548529261	0,000113921	0,021787032	Klf6
ENSMUSG00000078350	-0,299773339	4,939087289	0,000118743	0,022432279	Smim1
ENSMUSG00000026893	0,285399732	5,321880755	0,000141361	0,026383475	Gca
ENSMUSG00000029417	0,473233783	3,540927844	0,00014528	0,026792052	Cxcl9
ENSMUSG00000076563	-0,602749225	2,859759396	0,000147507	0,026872124	Igkv5-48
ENSMUSG00000046186	-0,449382679	5,013306583	0,000152015	0,026872124	Cd109
ENSMUSG00000026180	0,256301669	7,13398943	0,000153672	0,026872124	Cxcr2
ENSMUSG00000059895	-0,244325799	8,267757925	0,000154337	0,026872124	Ptp4a3
ENSMUSG00000026922	0,26096275	5,473764701	0,000154388	0,026872124	Agpat2
ENSMUSG00000021556	-0,265892627	5,585969641	0,000161271	0,027758389	Golm1
ENSMUSG00000095403	-0,857994803	1,793306924	0,00016649	0,028097533	Gm21092
ENSMUSG00000095497	2,851843306	3,378048768	0,000169973	0,028097533	Igkv1-122
ENSMUSG00000062593	0,272883335	5,490399256	0,000170265	0,028097533	Gm49339
ENSMUSG00000049130	0,290052414	5,690468584	0,000170805	0,028097533	C5ar1
ENSMUSG00000026980	0,29656023	5,235883099	0,000174013	0,028097533	Ly75
ENSMUSG00000038085	0,768271548	3,240690549	0,000174125	0,028097533	Cnbd2
ENSMUSG00000039910	0,32071959	5,745267933	0,000178754	0,02854726	Cited2
ENSMUSG00000024079	-0,300045024	5,384143958	0,000183149	0,028950576	Eif2ak2
ENSMUSG00000094075	-0,572484121	3,436768549	0,000187912	0,029403506	Ighv1-80
ENSMUSG00000038517	0,300684319	4,841077145	0,000190015	0,029435201	Tbkbp1
ENSMUSG00000051906	-0,973739937	2,035042527	0,000197998	0,030311172	Cd209f

ENSMUSG00000064354	0,447823739	8,270800455	0,000199583	0,030311172	mt-Co2
ENSMUSG00000038633	0,239370224	6,67889999	0,00021462	0,032278428	Degs1
ENSMUSG00000021054	0,298623829	5,433743028	0,000218238	0,032382575	Sgpp1
ENSMUSG00000103168	-0,715699403	2,286394034	0,000219493	0,032382575	Gm30948
ENSMUSG00000071713	0,245729276	7,056223194	0,000236269	0,034528715	Csf2rb
ENSMUSG00000076545	-0,40714524	4,014776008	0,000238706	0,034558857	Igkv4-72
ENSMUSG00000027508	0,2433919	6,166202138	0,00025057	0,035930083	Pag1
ENSMUSG00000112478	0,405614565	5,16175122	0,000252816	0,035930083	Gm47761
ENSMUSG00000076525	-1,736440775	0,63931903	0,000257788	0,036303564	Igkv1-99
ENSMUSG00000055435	0,264500143	5,742833925	0,000274842	0,038356556	Maf
ENSMUSG00000021775	0,361259771	4,611208753	0,000279129	0,038607055	Nr1d2
ENSMUSG00000018476	0,236114901	5,974330476	0,000295202	0,040250851	Kdm6b
ENSMUSG00000028382	0,255929686	8,101689878	0,000296211	0,040250851	Ptbp3
ENSMUSG00000052837	0,476965799	4,488704017	0,000304035	0,040954805	Junb
ENSMUSG00000054676	0,411623347	5,611112835	0,000317803	0,042440425	1600014C10Rik
ENSMUSG00000094694	0,686467059	4,428294267	0,000320616	0,042450055	Ighv1-9
ENSMUSG00000029413	0,299739296	5,086828562	0,000333663	0,043803131	Naaa
ENSMUSG00000037788	-0,268730019	6,812988599	0,00034251	0,044280987	Vopp1
ENSMUSG00000003746	0,245725223	6,053372984	0,00034302	0,044280987	Man1a
ENSMUSG00000069045	0,262988014	5,548985287	0,000360772	0,046187779	Ddx3y
ENSMUSG00000042045	-0,860592436	2,158643427	0,000368233	0,046756493	Sln
ENSMUSG00000060675	-0,214436487	6,362054623	0,000375714	0,047102351	Plaat3
ENSMUSG00000096805	1,145576998	1,924848216	0,000377038	0,047102351	Ighv9-1
ENSMUSG00000068587	0,268525917	5,094659463	0,000387656	0,048041425	Mgam
ENSMUSG00000052270	0,294501965	6,202327479	0,000413077	0,050785483	Fpr2
ENSMUSG00000072214	0,241274566	6,328101755	0,000417922	0,050976614	Septin5
ENSMUSG00000029530	0,511262144	2,953795065	0,000424968	0,051431113	Ccr9
ENSMUSG00000062210	0,243287342	6,106028214	0,000444867	0,053098981	Tnfaip8
ENSMUSG00000096719	0,28474539	5,39182391	0,000445605	0,053098981	Mrgpra2b
ENSMUSG00000076587	1,450397786	5,512605489	0,000450391	0,053259635	Igkv6-20
ENSMUSG00000120144	0,319210463	4,55658941	0,000493818	0,057952491	Gm56517
ENSMUSG00000002111	0,272545128	6,944985798	0,000500695	0,058317839	Spi1
ENSMUSG00000039452	-0,295007099	5,927263811	0,000522672	0,060044703	Snx22
ENSMUSG00000036181	1,038003819	8,336838034	0,000523274	0,060044703	H1f2

ENSMUSG00000007379	-0,311406435	4,676552192	0,000533127	0,060725476	Dennd2c
ENSMUSG00000047810	0,251530323	6,459887923	0,000537213	0,06074433	Ccdc88b
ENSMUSG00000038642	0,288195891	7,633418516	0,000554017	0,061816308	Ctss
ENSMUSG00000031246	0,320867587	6,283044506	0,000554675	0,061816308	Sh3bgrl
ENSMUSG00000024900	-0,254670799	6,764549094	0,000573244	0,063429495	Cpt1a
ENSMUSG00000044712	0,213444168	6,41450552	0,000578284	0,063437434	Slc38a6
ENSMUSG00000005057	0,530367918	3,619068101	0,000583874	0,063437434	Sh2b2
ENSMUSG00000032202	0,208467801	6,473586908	0,000585602	0,063437434	Rab27a
ENSMUSG00000025877	0,24914307	7,658865056	0,000593326	0,063827838	Hk3
ENSMUSG00000026872	0,221221697	6,745848474	0,000598177	0,063905895	Zeb2
ENSMUSG00000039477	0,270017349	6,631926521	0,000605488	0,064243898	Tnrc18
ENSMUSG00000076666	1,414526546	3,75979742	0,000615143	0,064504869	Ighv14-4
ENSMUSG00000026796	0,311657714	5,259145824	0,00062121	0,064504869	Niban2
ENSMUSG00000029304	-0,349795001	9,208617019	0,000624399	0,064504869	Spp1
ENSMUSG00000000318	0,476844402	3,73077231	0,000624603	0,064504869	Clec10a
ENSMUSG00000028626	0,961461447	1,890682454	0,000641601	0,065821458	Col9a2
ENSMUSG00000030144	0,425790111	4,683075224	0,000648376	0,066078857	Clec4d
ENSMUSG00000096577	4,291663111	1,404460483	0,000668055	0,067307275	Ighv1-71
ENSMUSG00000022698	0,202610515	6,632438524	0,000669119	0,067307275	Naa50
ENSMUSG00000022971	0,312273013	6,161508703	0,000677541	0,0677148	Ifnar2
ENSMUSG00000001444	0,816002134	1,506802631	0,000696711	0,068841765	Tbx21
ENSMUSG00000095981	-0,805021858	1,896952254	0,000700044	0,068841765	Ighv10-1
ENSMUSG00000002257	0,245006125	5,704950636	0,00070215	0,068841765	Def6
ENSMUSG00000026005	0,31482288	4,935014028	0,00074415	0,072500778	Rpe
ENSMUSG00000071076	0,441823566	4,923940806	0,000773031	0,074708904	Jund
ENSMUSG00000036686	0,281666564	4,568743249	0,000776459	0,074708904	Cc2d1a
ENSMUSG00000022324	0,58234155	2,441221974	0,000787301	0,075284476	Matn2
ENSMUSG00000013974	0,214474701	6,497393964	0,000794704	0,075526169	Mcemp1
ENSMUSG00000046516	-0,246544616	5,209774318	0,000805106	0,076048106	Cox17
ENSMUSG00000121605	-0,286531548	9,449512266	0,000814242	0,076444941	Ahsp
ENSMUSG00000073421	0,274331474	7,723833095	0,00083681	0,07809048	H2-Ab1
ENSMUSG00000030341	0,274695853	6,589401635	0,000853722	0,079191637	Tnfrsf1a
ENSMUSG00000029086	0,282498964	4,798768898	0,000859279	0,079232652	Prom1
ENSMUSG00000041836	0,208189719	6,237226441	0,000867859	0,079550319	Ptpre

ENSMUSG00000036282	0,32847477	4,149366346	0,000875362	0,079766069	Naa30
ENSMUSG00000045817	0,240685074	7,125693444	0,000883493	0,080036243	Zfp36l2
ENSMUSG00000030589	0,237523592	6,456232179	0,000906626	0,081589131	Rasgrp4
ENSMUSG00000051504	0,318260175	4,535512657	0,000911169	0,081589131	Siglech
ENSMUSG00000063406	0,287440086	4,73401451	0,000918928	0,081810991	Tmed5
ENSMUSG00000050921	0,567056769	2,859947892	0,000935559	0,082453725	P2ry10
ENSMUSG00000026158	0,252883047	6,176748181	0,000936793	0,082453725	Ogfrl1
ENSMUSG00000015312	0,51137912	3,462856262	0,000943746	0,082596398	Gadd45b
ENSMUSG00000028161	0,211906984	6,525630971	0,000950977	0,08276167	Ppp3ca
ENSMUSG00000032328	0,221520984	6,475141369	0,000988299	0,085529249	Tmem30a
ENSMUSG00000020948	0,385838232	3,860396002	0,001003289	0,085604057	Klhl28
ENSMUSG00000048047	0,297579224	4,347609668	0,001009428	0,085604057	Zbtb33
ENSMUSG00000076615	0,925272317	5,715986109	0,001011753	0,085604057	Ighg3
ENSMUSG00000043832	0,344709269	4,244977815	0,001021997	0,085604057	Clec4a3
ENSMUSG00000040659	0,234720039	6,805711876	0,001024024	0,085604057	Efhd2
ENSMUSG00000022358	-0,462234673	5,403454468	0,001029104	0,085604057	Fbxo32
ENSMUSG00000025795	0,21224157	6,229883999	0,00103409	0,085604057	Rassf3
ENSMUSG00000023274	0,273363863	5,646817613	0,001039042	0,085604057	Cd4
ENSMUSG00000036928	-0,659344043	2,333597215	0,001042356	0,085604057	Stag3
ENSMUSG00000068417	-0,275629691	4,671828211	0,001044424	0,085604057	Pnp2
ENSMUSG00000022858	0,212320657	7,023993419	0,001066318	0,086938604	Tra2b
ENSMUSG00000030470	-0,607129389	3,855430697	0,001082265	0,087248289	Csrp3
ENSMUSG00000034993	0,205616977	6,29764997	0,001085289	0,087248289	Vat1
ENSMUSG00000024679	0,44376443	3,233803451	0,001087013	0,087248289	Ms4a6d
ENSMUSG00000035493	0,268013473	7,244977015	0,001098043	0,087679337	Tgfbi
ENSMUSG00000062949	0,262460543	5,122207238	0,00112882	0,089674595	Atp11c
ENSMUSG00000020132	0,201883435	6,26764611	0,00113462	0,089675539	Rab21
ENSMUSG00000118491	-1,356717229	-0,183246354	0,001146061	0,08988854	Gm44505
ENSMUSG00000076569	-0,671035182	5,147820925	0,001148921	0,08988854	Igkv5-39
ENSMUSG00000055805	0,231110837	7,784041605	0,001206785	0,0939412	Fmnl1
ENSMUSG00000032744	0,552444052	2,895374594	0,001219496	0,094332982	Heyl
ENSMUSG00000023903	0,216220297	6,478059874	0,001223996	0,094332982	Mmp25
ENSMUSG00000066366	0,912763236	2,754691569	0,001251945	0,09600052	Serpina1a
ENSMUSG00000026483	0,20116641	6,828646483	0,001258028	0,09600052	Niban1

ENSMUSG00000079227	0,496915376	2,983345677	0,001281755	0,097331728	Ccr5
ENSMUSG00000021033	-0,335070064	4,260381013	0,001297034	0,09801149	Gstz1
ENSMUSG00000039396	-0,222017583	5,941835595	0,00131156	0,098515905	Neil3
ENSMUSG00000024260	-0,217095747	5,677509094	0,001316428	0,098515905	Sap130
ENSMUSG00000076940	0,569343547	2,668727348	0,001323382	0,098560113	Iglv2
ENSMUSG00000075511	-0,414531387	3,480834461	0,00133528	0,098595577	1700001L05Rik
ENSMUSG00000095200	-0,843071218	3,784187282	0,001336587	0,098595577	Ighv1-7

Table S3

ID	Description	GeneRatio	BgRatio	RichFactor	FoldEnrichment	zScore	pvalue	p.adjust	qvalue	geneID	Count
GO:0016064	immunoglobulin mediated immune response	12/103	260/28832	0.0461538461538462	12.9194921583271	11.5601193066537	1.65877507637859e-10	1.31343008676197e-07	1.0407760732154e-07	Ighv9-4/Ighv1-77/Ighv9-3/Trem2/Ighv1-82/Ighv1-63/Ighv1-64/Ighv1-12/Fgl2/Ighv9-2/Csf2rb/Ighv9-1	12
GO:0019724	B cell mediated immunity	12/103	264/28832	0.0454545454545455	12.7237422771403	11.4582031631078	1.97691118953853e-10	1.31343008676197e-07	1.0407760732154e-07	Ighv9-4/Ighv1-77/Ighv9-3/Trem2/Ighv1-82/Ighv1-63/Ighv1-64/Ighv1-12/Fgl2/Ighv9-2/Csf2rb/Ighv9-1	12
GO:0002460	adaptive immune response based on somatic recombination of immune receptors built from immunoglobulin superfamily domains	15/103	487/28832	0.0308008213552361	8.62183768266183	10.1572035628866	2.1988226898917e-10	1.31343008676197e-07	1.0407760732154e-07	Ighv9-4/Ighv1-77/Ighv9-3/Trem2/Ccr2/Ighv1-82/Ighv1-63/Ighv1-64/Ighv1-12/Fgl2/Ighv9-2/Sema4a/Lilrb4a/Csf2rb/Ighv9-1	15
GO:0002449	lymphocyte mediated immunity	14/103	477/28832	0.0293501048218029	8.21574973031284	9.51511488411409	1.71168080538931e-09	7.66833000814413e-07	6.07646685913206e-07	Ighv9-4/Ighv1-77/Ighv9-3/Trem2/Ccr2/Ighv1-82/Ighv1-63/Ighv1-64/Ighv1-12/Fgl2/Ighv9-2/Lilrb4a/Csf2rb/Ighv9-1	14
GO:0097529	myeloid leukocyte migration	8/103	255/28832	0.0313725490196078	8.78187702265372	7.4736773867421	3.8661870320309e-06	0.00138564143227988	0.00109799711709678	Ccl3/Trem2/Ccr2/Prtn3/Kitl/Cxcl9/Cxcr2/C5ar1	8
GO:0070098	chemokine-mediated signaling pathway	5/103	76/28832	0.0657894736842105	18.4159427695452	9.10285779689455	7.96955808979027e-06	0.00238024134948403	0.00188612874791703	Ccl3/Trem2/Ccr2/Cxcl9/Cxcr2	5
GO:0002437	inflammatory response to antigenic stimulus	5/103	81/28832	0.0617283950617284	17.2791561788326	8.78488605006079	1.08974114693745e-05	0.00250911529400183	0.0019882498423452	Trem2/Ccr2/Cxcr2/Lilrb4a/Kdm6b	5
GO:0002573	myeloid leukocyte differentiation	8/103	295/28832	0.0271186440677966	7.59111403653118	6.81324312705181	1.12014075625082e-05	0.00250911529400183	0.0019882498423452	Ccl3/Trem2/Prtn3/Kitl/Cd109/Lilrb4a/Cited2/Junb	8
GO:1990868	response to chemokine	5/103	87/28832	0.0574712643678161	16.0874902354648	8.43885806780859	1.54514252614028e-05	0.00255147359642301	0.0020218150150227	Ccl3/Trem2/Ccr2/Cxcl9/Cxcr2	5
GO:1990869	cellular response to chemokine	5/103	87/28832	0.0574712643678161	16.0874902354648	8.43885806780859	1.54514252614028e-05	0.00255147359642301	0.0020218150150227	Ccl3/Trem2/Ccr2/Cxcl9/Cxcr2	5
GO:0019852	L-ascorbic acid metabolic process	3/103	14/28832	0.214285714285714	59.9833564493759	13.2175144093039	1.56619472994716e-05	0.00255147359642301	0.0020218150150227	Ugt1a6a/Atp1a3/Ero1a	3
GO:0002262	myeloid cell homeostasis	7/103	233/28832	0.0300429184549356	8.40968373682237	6.79971043992461	2.10992741197408e-05	0.00315082493521463	0.00249674743750266	Ccr2/Klf2/Kitl/Cxcr2/Lilrb4a/Cited2/Ptbp3	7
GO:1901334	lactone metabolic process	3/103	17/28832	0.176470588235294	49.3980582524272	11.9517444184344	2.90312239792005e-05	0.00400184256697902	0.00317110292695882	Ugt1a6a/Atp1a3/Ero1a	3
GO:0002687	positive regulation of leukocyte migration	6/103	180/28832	0.0333333333333333	9.33074433656958	6.71321874560718	4.71706024769372e-05	0.00603783711704797	0.00478444682266078	Ccl3/Trem2/Ccr2/Kitl/Cxcr2/C5ar1	6
GO:0043117	positive regulation of vascular permeability	3/103	21/28832	0.142857142857143	39.9889042995839	10.7018712617499	5.61944162576528e-05	0.00671335959558092	0.00531973807239113	Fgfbp3/Ptp4a3/Cxcr2	3
GO:1990266	neutrophil migration	5/103	129/28832	0.0387596899224806	10.8497027169414	6.71340029391801	0.000102234992591984	0.0110595574728436	0.0087637118367399	Ccl3/Prtn3/Cxcl9/Cxcr2/C5ar1	5
GO:0050900	leukocyte migration	8/103	408/28832	0.0196078431372549	5.48867313915858	5.46756149934871	0.000110675351655043	0.0110595574728436	0.0087637118367399	Ccl3/Trem2/Ccr2/Prtn3/Kitl/Cxcl9/Cxcr2/C5ar1	8
GO:1905517	macrophage migration	4/103	69/28832	0.0579710144927536	16.2273814549036	7.58264794674717	0.000111089304972759	0.0110595574728436	0.0087637118367399	Ccl3/Trem2/Ccr2/C5ar1	4
GO:0030316	osteoclast differentiation	5/103	137/28832	0.0364963503649635	10.2161434342003	6.47432661817875	0.000135668154570357	0.012795649104741	0.0101394094468372	Ccl3/Trem2/Cd109/Lilrb4a/Junb	5
GO:0008347	glial cell migration	4/103	75/28832	0.0533333333333333	14.9291909385113	7.23224192711832	0.000153644873441682	0.0130694767441499	0.0103563934021724	Ccl3/Vcan/Trem2/Ccr2	4
GO:0045765	regulation of angiogenesis	7/103	320/28832	0.021875	6.12330097087379	5.51822276092249	0.000154867849169061	0.0130694767441499	0.0103563934021724	Ccr2/Klf2/Lrg1/Hhip/Sema4a/Cxcr2/C5ar1	7
GO:0002286	T cell activation involved in immune response	5/103	142/28832	0.0352112676056338	9.85642007384111	6.33468926659933	0.000160451165385769	0.0130694767441499	0.0103563934021724	Trem2/Ccr2/Fgl2/Clec4e/Sema4a	5
GO:0048872	homeostasis of number of cells	8/103	436/28832	0.018348623853211	5.13618954306582	5.21078679553588	0.000174210613604502	0.013203111743736	0.0104622872076479	Ccr2/Klf2/Kitl/Pianp/Cxcr2/Lilrb4a/Cited2/Ptbp3	8
GO:1901342	regulation of vasculature development	7/103	327/28832	0.0214067278287462	5.99222113357679	5.43619940704955	0.000176827389425035	0.013203111743736	0.0104622872076479	Ccr2/Klf2/Lrg1/Hhip/Sema4a/Cxcr2/C5ar1	7
GO:0002366	leukocyte activation involved in immune response	7/103	341/28832	0.0205278592375367	5.74620618967628	5.27907892826899	0.000228313615069992	0.0148398946713287	0.0117592915364323	Ccl3/Trem2/Ccr2/Fgl2/Clec4e/Sema4a/Lilrb4a	7
GO:0001666	response to hypoxia	7/103	342/28832	0.0204678362573099	5.72940441719185	5.26819075434063	0.000232409825448111	0.0148398946713287	0.0117592915364323	Ucp3/Trem2/Ccr2/Ero1a/Cpeb2/Cited2/mt-Co2	7
GO:0002285	lymphocyte activation involved in immune response	6/103	241/28832	0.024896265560166	6.96902066631753	5.57166686677218	0.000233066173108574	0.0148398946713287	0.0117592915364323	Trem2/Ccr2/Fgl2/Clec4e/Sema4a/Lilrb4a	6
GO:0001780	neutrophil homeostasis	3/103	34/28832	0.0882352941176471	24.6990291262136	8.27898432442064	0.000244443364917105	0.0148398946713287	0.0117592915364323	Ccr2/Cxcr2/Lilrb4a	3
GO:0002263	cell activation involved in immune response	7/103	345/28832	0.0202898550724638	5.67958350921626	5.23578256213761	0.000245055576630879	0.0148398946713287	0.0117592915364323	Ccl3/Trem2/Ccr2/Fgl2/Clec4e/Sema4a/Lilrb4a	7
GO:1902600	proton transmembrane transport	5/103	156/28832	0.032051282051282	8.97186955439382	5.9779380169808	0.00024843573668519	0.0148398946713287	0.0117592915364323	mt-Atp8/Ucp3/mt-Co3/Atp1a3/mt-Co2	5
GO:0002292	T cell differentiation involved in immune response	4/103	90/28832	0.0444444444444444	12.4409924487594	6.50900600041285	0.000310032406975367	0.0179218733322535	0.0142014844485491	Ccr2/Fgl2/Clec4e/Sema4a	4
GO:0002685	regulation of leukocyte migration	6/103	257/28832	0.0233463035019455	6.53515167541838	5.3369259732211	0.000328687734353205	0.0184065131237795	0.0145855182119235	Ccl3/Trem2/Ccr2/Kitl/Cxcr2/C5ar1	6
GO:0097530	granulocyte migration	5/103	169/28832	0.029585798816568	8.28172574251738	5.684667386729	0.00035919508138827	0.0194506326425136	0.0154128897055632	Ccl3/Prtn3/Cxcl9/Cxcr2/C5ar1	5
GO:0050921	positive regulation of chemotaxis	5/103	170/28832	0.0294117647058824	8.23300970873786	5.66341613330651	0.000369041021119119	0.0194506326425136	0.0154128897055632	Ccl3/Trem2/Ccr2/Cxcr2/C5ar1	5
GO:1902105	regulation of leukocyte differentiation	7/103	373/28832	0.0187667560321716	5.25323407688904	4.95053452475913	0.000391991524833397	0.0200699660714699	0.0159036561503835	Cyp26b1/Ccl3/Trem2/Ccr2/Fgl2/Kitl/Lilrb4a	7
GO:1990845	adaptive thermogenesis	5/103	175/28832	0.0285714285714286	7.99778085991678	5.55971092746648	0.000421367169519433	0.0203324964333416	0.016111688021956	Ucp3/Fabp5/Ccr2/Kdm6b/Sln	5
GO:0036293	response to decreased oxygen levels	7/103	378/28832	0.0185185185185185	5.18374685364977	4.90261563428434	0.000424421500902787	0.0203324964333416	0.016111688021956	Ucp3/Trem2/Ccr2/Ero1a/Cpeb2/Cited2/mt-Co2	7
GO:0070661	leukocyte proliferation	7/103	379/28832	0.0184696569920844	5.17006942131824	4.89313354418419	0.000431157848474879	0.0203324964333416	0.016111688021956	Trem2/Ccr2/Gpnmb/Kitl/Lilrb4a/Csf2rb/Junb	7
GO:0006766	vitamin metabolic process	4/103	99/28832	0.0404040404040404	11.3099931352359	6.15281274189938	0.000445667175196723	0.0204778353321161	0.0162268561225473	Cyp26b1/Ugt1a6a/Atp1a3/Ero1a	4
GO:0030593	neutrophil chemotaxis	4/103	100/28832	0.04	11.1968932038835	6.11607993219135	0.000462972118668541	0.0207411509163506	0.0164355102127332	Ccl3/Cxcl9/Cxcr2/C5ar1	4
GO:0120162	positive regulation of cold-induced thermogenesis	4/103	101/28832	0.0396039603960396	11.0860328751322	6.07986457790818	0.000480750795930165	0.0210123274708989	0.0166503934200204	Fabp5/Ccr2/Kdm6b/Sln	4
GO:0050863	regulation of T cell activation	7/103	391/28832	0.0179028132992327	5.01139721401435	4.78189269977346	0.000518872482581628	0.0221385592568161	0.0175428315539503	Cyp26b1/Ccr2/Gpnmb/Fgl2/Kitl/Lilrb4a/Pag1	7
GO:0002274	myeloid leukocyte activation	6/103	282/28832	0.0212765957446809	5.95579425738484	5.0075232765788	0.000536895600538123	0.0222755710602865	0.0176514011750038	Ccl3/Trem2/Ccr2/Cxcr2/Lilrb4a/C5ar1	6
GO:0060136	embryonic process involved in female pregnancy	2/103	10/28832	0.2	55.9844660194175	10.4127673603762	0.00055821188865833	0.0222755710602865	0.0176514011750038	Cited2/Junb	2
GO:0030522	intracellular receptor signaling pathway	7/103	396/28832	0.0176767676767677	4.94812199666569	4.73687707661347	0.000559375389348711	0.0222755710602865	0.0176514011750038	Cyp26b1/Trem2/Fabp5/Klf2/Cited2/Eif2ak2/Nr1d2	7
GO:0071677	positive regulation of mononuclear cell migration	4/103	107/28832	0.0373831775700935	10.4643861718537	5.87276378901567	0.000597785292448755	0.0232876357406124	0.0184533720712442	Ccl3/Trem2/Ccr2/C5ar1	4
GO:0002003	angiotensin maturation	2/103	11/28832	0.181818181818182	50.8949691085613	9.91029970898038	0.0006806689452823	0.0248930357131813	0.0197255082102218	Anpep/Cpa3	2
GO:0035360	positive regulation of peroxisome proliferator activated receptor signaling pathway	2/103	11/28832	0.181818181818182	50.8949691085613	9.91029970898038	0.0006806689452823	0.0248930357131813	0.0197255082102218	Fabp5/Cited2	2
GO:0043615	astrocyte cell migration	2/103	11/28832	0.181818181818182	50.8949691085613	9.91029970898038	0.0006806689452823	0.0248930357131813	0.0197255082102218	Ccl3/Ccr2	2
GO:0043114	regulation of vascular permeability	3/103	49/28832	0.0612244897959184	17.1381018426788	6.76971949184966	0.000723930404982062	0.0254369271711344	0.0201564936289123	Fgfbp3/Ptp4a3/Cxcr2	3
GO:1905521	regulation of macrophage migration	3/103	49/28832	0.0612244897959184	17.1381018426788	6.76971949184966	0.000723930404982062	0.0254369271711344	0.0201564936289123	Trem2/Ccr2/C5ar1	3
GO:0032680	regulation of tumor necrosis factor production	5/103	200/28832	25	6.99805825242719	5.09668796025863	0.000771418059561555	0.0260022018171131	0.0206044233148999	Ccl3/Trem2/Ccr2/Gpnmb/Lilrb4a	5
GO:0002275	myeloid cell activation involved in immune response	4/103	115/28832	0.0347826086956522	9.73642887294217	5.62084413194406	0.000783548492256755	0.0260022018171131	0.0206044233148999	Ccl3/Trem2/Ccr2/Lilrb4a	4
GO:0002690	positive regulation of leukocyte chemotaxis	4/103	115/28832	0.0347826086956522	9.73642887294217	5.62084413194406	0.000783548492256755	0.0260022018171131	0.0206044233148999	Ccl3/Ccr2/Cxcr2/C5ar1	4
GO:1903555	regulation of tumor necrosis factor superfamily cytokine production	5/103	203/28832	0.0246305418719212	6.89463867234205	5.04650060242744	0.000824690534519369	0.0267482305543721	0.021195584479469	Ccl3/Trem2/Ccr2/Gpnmb/Lilrb4a	5
GO:0006767	water-soluble vitamin metabolic process	3/103	52/28832	0.0576923076923077	16.1493651979089	6.54694847179997	0.000861658221883812	0.0267482305543721	0.021195584479469	Ugt1a6a/Atp1a3/Ero1a	3
GO:0016525	negative regulation of angiogenesis	4/103	118/28832	0.0338983050847458	9.48889254566398	5.53265261587974	0.000862665569116953	0.0267482305543721	0.021195584479469	Ccr2/Klf2/Hhip/Sema4a	4
GO:0007159	leukocyte cell-cell adhesion	7/103	427/28832	0.0163934426229508	4.5888906573293	4.47368064206839	0.000870124017149109	0.0267482305543721	0.021195584479469	Ccr2/Lrg1/Gpnmb/Fgl2/Kitl/Lilrb4a/Pag1	7
GO:0032640	tumor necrosis factor production	5/103	206/28832	0.0242718446601942	6.7942313130361	4.99732179702996	0.000880661608653992	0.0267482305543721	0.021195584479469	Ccl3/Trem2/Ccr2/Gpnmb/Lilrb4a	5
GO:2000181	negative regulation of blood vessel morphogenesis	4/103	120/28832	0.0333333333333333	9.33074433656958	5.47558994931877	0.000918438563220563	0.0273299906051294	0.0216565773768324	Ccr2/Klf2/Hhip/Sema4a	4
GO:0071706	tumor necrosis factor superfamily cytokine production	5/103	209/28832	0.0239234449760766	6.69670646165281	4.94911585399341	0.000939416311880867	0.0273299906051294	0.0216565773768324	Ccl3/Trem2/Ccr2/Gpnmb/Lilrb4a	5
GO:1901343	negative regulation of vasculature development	4/103	121/28832	0.0330578512396694	9.25363074701115	5.44755685671362	0.000947256130815318	0.0273299906051294	0.0216565773768324	Ccr2/Klf2/Hhip/Sema4a	4
GO:0002291	T cell activation via T cell receptor contact with antigen bound to MHC molecule on antigen presenting cell	2/103	13/28832	0.153846153846154	43.0649738610904	9.08324559229308	0.00096081998221158	0.0273299906051294	0.0216565773768324	Trem2/Fgl2	2
GO:0036230	granulocyte activation	3/103	56/28832	0.0535714285714286	14.995839112344	6.27719981532471	0.00106965788101457	0.0292477568937538	0.0231762359314343	Ccl3/Ccr2/Cxcr2	3
GO:0070482	response to oxygen levels	7/103	444/28832	0.0157657657657658	4.41318988891804	4.33983030286632	0.00109082084821621	0.0292477568937538	0.0231762359314343	Ucp3/Trem2/Ccr2/Ero1a/Cpeb2/Cited2/mt-Co2	7

GO:0002002	regulation of angiotensin levels in blood	2/103	14/28832	0.142857142857143	39.9889042995839	8.73697997883562	0.0011183463213636	0.0292477568937538	0.0231762359314343	Anpep/Cpa3	2
GO:0048385	regulation of retinoic acid receptor signaling pathway	2/103	14/28832	0.142857142857143	39.9889042995839	8.73697997883562	0.0011183463213636	0.0292477568937538	0.0231762359314343	Cyp26b1/Klf2	2
GO:1903977	positive regulation of glial cell migration	2/103	14/28832	0.142857142857143	39.9889042995839	8.73697997883562	0.0011183463213636	0.0292477568937538	0.0231762359314343	Trem2/Ccr2	2
GO:0048821	erythrocyte development	3/103	57/28832	0.0526315789473684	14.7327542156362	6.21406253855913	0.0011261692107528	0.0292477568937538	0.0231762359314343	Klf2/Cited2/Ptbp3	3
GO:1902107	positive regulation of leukocyte differentiation	5/103	221/28832	0.0226244343891403	6.33308439133682	4.76536625335746	0.00120399868534693	0.0303882485090381	0.0240799737069387	Ccl3/Trem2/Ccr2/Kitl/Lilrb4a	5
GO:1903708	positive regulation of hemopoiesis	5/103	221/28832	0.0226244343891403	6.33308439133682	4.76536625335746	0.00120399868534693	0.0303882485090381	0.0240799737069387	Ccl3/Trem2/Ccr2/Kitl/Lilrb4a	5
GO:0032494	response to peptidoglycan	2/103	15/28832	0.133333333333333	37.3229773462783	8.42540714883791	0.00128739551161328	0.0317571613554026	0.0251647149133212	Trem2/C5ar1	2
GO:1903706	regulation of hemopoiesis	7/103	459/28832	0.0152505446623094	4.26896799712334	4.22719802850313	0.0013201386867282	0.0317571613554026	0.0251647149133212	Cyp26b1/Ccl3/Trem2/Ccr2/Fgl2/Kitl/Lilrb4a	7
GO:0090287	regulation of cellular response to growth factor stimulus	6/103	336/28832	0.0178571428571429	4.99861303744799	4.4144365204489	0.00132799327965145	0.0317571613554026	0.0251647149133212	Fgfbp3/Lrg1/Hhip/Cd109/Ptp4a3/Cited2	6
GO:0050866	negative regulation of cell activation	5/103	226/28832	0.0221238938053097	6.19297190480282	4.69277612512494	0.00132912226654866	0.0317571613554026	0.0251647149133212	Trem2/Gpnmb/Fgl2/Lilrb4a/Pag1	5
GO:0070374	positive regulation of ERK1 and ERK2 cascade	5/103	227/28832	0.0220264317180617	6.16569009024422	4.67852022126295	0.00135525661957644	0.0319555245036972	0.0253218999973493	Ccl3/Trem2/Fgfbp3/Gpnmb/C5ar1	5
GO:0071621	granulocyte chemotaxis	4/103	136/28832	0.0294117647058824	8.23300970873786	5.06251159760455	0.00145942525588588	0.0337237129363924	0.0267230314562931	Ccl3/Cxcl9/Cxcr2/C5ar1	4
GO:0006968	cellular defense response	2/103	16/28832	125	34.9902912621359	8.14303385504115	0.00146788482647244	0.0337237129363924	0.0267230314562931	Ccr2/Cxcr2	2
GO:0030217	T cell differentiation	6/103	348/28832	0.0172413793103448	4.82624707063944	4.29982078068607	0.00158652211777612	0.0355714491437517	0.0281871974241783	Cyp26b1/Ccr2/Fgl2/Clec4e/Sema4a/Lilrb4a	6
GO:0006935	chemotaxis	7/103	475/28832	0.0147368421052632	4.12517118037813	4.11224219179173	0.00160471170294402	0.0355714491437517	0.0281871974241783	Ccl3/Trem2/Ccr2/Sema4a/Cxcl9/Cxcr2/C5ar1	7
GO:0050920	regulation of chemotaxis	5/103	236/28832	0.0211864406779661	5.93055784103999	4.55394337284213	0.00160786126152003	0.0355714491437517	0.0281871974241783	Ccl3/Trem2/Ccr2/Cxcr2/C5ar1	5
GO:0042330	taxis	7/103	477/28832	0.0146750524109015	4.10787486515642	4.09822783392625	0.00164340383046544	0.0359143861487081	0.0284589443812308	Ccl3/Trem2/Ccr2/Sema4a/Cxcl9/Cxcr2/C5ar1	7
GO:0043603	amide metabolic process	7/103	479/28832	0.0146137787056367	4.09072298680503	4.08428971423997	0.00168281976249605	0.0363326869203967	0.0287904103945108	Pdk4/Trem2/Anpep/Cpa3/Degs1/Naaa/Plaat3	7
GO:0002688	regulation of leukocyte chemotaxis	4/103	143/28832	0.027972027972028	7.82999524747098	4.90251493885571	0.00175414014023837	0.0369814015447901	0.029304458813394	Ccl3/Ccr2/Cxcr2/C5ar1	4
GO:0050868	negative regulation of T cell activation	4/103	143/28832	0.027972027972028	7.82999524747098	4.90251493885571	0.00175414014023837	0.0369814015447901	0.029304458813394	Gpnmb/Fgl2/Lilrb4a/Pag1	4
GO:0030595	leukocyte chemotaxis	5/103	242/28832	0.0206611570247934	5.78351921688197	4.47441579131556	0.00179446842302229	0.0373917141169296	0.0296295948917634	Ccl3/Ccr2/Cxcl9/Cxcr2/C5ar1	5
GO:0019722	calcium-mediated signaling	5/103	243/28832	0.0205761316872428	5.75971872627752	4.46142045606559	0.00182704880850671	0.0375788963577366	0.0297779201049029	Ccl3/Trem2/Ccr2/Atp1a3/Cxcr2	5
GO:0001776	leukocyte homeostasis	4/103	145/28832	0.0275862068965517	7.7219953130231	4.85878688409589	0.00184539223185314	0.0375788963577366	0.0297779201049029	Ccr2/Kitl/Cxcr2/Lilrb4a	4
GO:0070371	ERK1 and ERK2 cascade	6/103	364/28832	0.0164835164835165	4.61410434225968	4.15490512016204	0.00198885292647866	0.0400452184747164	0.0317322601752775	Ccl3/Trem2/Fgfbp3/Atp1a3/Gpnmb/C5ar1	6
GO:0000768	syncytium formation by plasma membrane fusion	3/103	70/28832	0.0428571428571429	11.9966712898752	5.51555136205653	0.0020373210689429	0.0401195533576447	0.0317911639329551	Trem2/Cxcl9/Cd109	3
GO:0140253	cell-cell fusion	3/103	70/28832	0.0428571428571429	11.9966712898752	5.51555136205653	0.0020373210689429	0.0401195533576447	0.0317911639329551	Trem2/Cxcl9/Cd109	3
GO:1902033	regulation of hematopoietic stem cell proliferation	2/103	19/28832	0.105263157894737	29.4655084312724	7.43172647223193	0.00207717275213734	0.0404597127372838	0.0320607098699458	Kitl/Eif2ak2	2
GO:0032720	negative regulation of tumor necrosis factor production	3/103	71/28832	0.0422535211267606	11.8277040886093	5.46955221209872	0.00212172457153608	0.0407742645035864	0.0323099640597615	Trem2/Gpnmb/Lilrb4a	3
GO:0120161	regulation of cold-induced thermogenesis	4/103	151/28832	0.0264900662251656	7.4151610621745	4.73246165875072	0.00213882860677295	0.0407742645035864	0.0323099640597615	Fabp5/Ccr2/Kdm6b/Sln	4
GO:0006949	syncytium formation	3/103	72/28832	0.0416666666666667	11.663430420712	5.42446557759932	0.00220827258111198	0.041221088180757	0.032664031928948	Trem2/Cxcl9/Cd109	3
GO:1901224	positive regulation of non-canonical NF-kappaB signal transduction	3/103	72/28832	0.0416666666666667	11.663430420712	5.42446557759932	0.00220827258111198	0.041221088180757	0.032664031928948	Trem2/Ptp4a3/Eif2ak2	3
GO:0002761	regulation of myeloid leukocyte differentiation	4/103	154/28832	0.025974025974026	7.27070987265162	4.67187106631252	0.00229697919212506	0.0412626628025817	0.0326969761047243	Ccl3/Trem2/Kitl/Lilrb4a	4
GO:0035176	social behavior	3/103	73/28832	0.0410958904109589	11.5036574012502	5.38026043266493	0.00229698386102458	0.0412626628025817	0.0326969761047243	Trem2/Atp1a3/Pianp	3
GO:1903556	negative regulation of tumor necrosis factor superfamily cytokine production	3/103	73/28832	0.0410958904109589	11.5036574012502	5.38026043266493	0.00229698386102458	0.0412626628025817	0.0326969761047243	Trem2/Gpnmb/Lilrb4a	3
GO:0035358	regulation of peroxisome proliferator activated receptor signaling pathway	2/103	20/28832	0.1	27.9922330097087	7.2302833913183	0.00230260395103692	0.0412626628025817	0.0326969761047243	Fabp5/Cited2	2
GO:0071675	regulation of mononuclear cell migration	4/103	155/28832	0.0258064516129032	7.22380206702161	4.65203500708111	0.00235143823009796	0.0413115422385838	0.0327357086935207	Ccl3/Trem2/Ccr2/C5ar1	4
GO:0106106	cold-induced thermogenesis	4/103	155/28832	0.0258064516129032	7.22380206702161	4.65203500708111	0.00235143823009796	0.0413115422385838	0.0327357086935207	Fabp5/Ccr2/Kdm6b/Sln	4
GO:0003018	vascular process in circulatory system	5/103	259/28832	0.0193050193050193	5.40390598643026	4.26283194224227	0.00240938603383585	0.0419186385692606	0.0332167783305525	Fabp5/Fgfbp3/Klf2/Ptp4a3/Cxcr2	5
GO:0002577	regulation of antigen processing and presentation	2/103	21/28832	0.0952380952380952	26.6592695330559	7.0430858252212	0.00253906811259264	0.0425234584837945	0.0336960441110425	Trem2/Fgl2	2
GO:0006670	sphingosine metabolic process	2/103	21/28832	0.0952380952380952	26.6592695330559	7.0430858252212	0.00253906811259264	0.0425234584837945	0.0336960441110425	Sgpp1/Naaa	2
GO:0048143	astrocyte activation	2/103	21/28832	0.0952380952380952	26.6592695330559	7.0430858252212	0.00253906811259264	0.0425234584837945	0.0336960441110425	Trem2/C5ar1	2
GO:0140131	positive regulation of lymphocyte chemotaxis	2/103	21/28832	0.0952380952380952	26.6592695330559	7.0430858252212	0.00253906811259264	0.0425234584837945	0.0336960441110425	Ccl3/Ccr2	2
GO:0051703	biological process involved in intraspecies interaction between organisms	3/103	76/28832	0.0394736842105263	11.0495656617271	5.25264513955711	0.002576280799751	0.0427471777143869	0.0338733216263557	Trem2/Atp1a3/Pianp	3
GO:1903038	negative regulation of leukocyte cell-cell adhesion	4/103	160/28832	25	6.99805825242719	4.55543534750742	0.00263712413493436	0.043355288530297	0.0343551951523559	Gpnmb/Fgl2/Lilrb4a/Pag1	4
GO:0071456	cellular response to hypoxia	4/103	161/28832	0.0248447204968944	6.95459205210155	4.5366130925282	0.00269698959520519	0.0435413375096709	0.0345026223569937	Trem2/Ero1a/Cpeb2/Cited2	4
GO:1903037	regulation of leukocyte cell-cell adhesion	6/103	387/28832	0.0155038759689922	4.33988108677655	3.96069799627137	0.00269703597297627	0.0435413375096709	0.0345026223569937	Ccr2/Gpnmb/Fgl2/Kitl/Lilrb4a/Pag1	6
GO:0002763	positive regulation of myeloid leukocyte differentiation	3/103	78/28832	0.0384615384615385	10.7662434652726	5.17146898731148	0.00277362669438588	0.0443780271101741	0.0351656241609639	Ccl3/Trem2/Kitl	3
GO:0046519	sphingoid metabolic process	2/103	23/28832	0.0869565217391304	24.3410721823554	6.70515526164821	0.00304477513725655	0.0482852835925995	0.0382617760611	Sgpp1/Naaa	2
